# The economic impact of untreated maternal mental health conditions in Texas

**DOI:** 10.1186/s12884-022-05001-6

**Published:** 2022-09-12

**Authors:** Caroline Margiotta, Jessica Gao, So O’Neil, Divya Vohra, Kara Zivin

**Affiliations:** 1 Mathematica, 220 East Huron Street, Suite 300, Ann Arbor, MI 48104 USA; 2 University of Michigan Medical School, Ann Arbor, USA; 3 VA Ann Arbor Healthcare System, Ann Arbor, USA

**Keywords:** Maternal mental health conditions, Cost-of-illness, Health care payers, Texas

## Abstract

**Background:**

Maternal mental health conditions (MMHCs), which include depression and anxiety disorders during pregnancy and through five years postpartum, are among the most common obstetric complications in the United States overall and in Texas in particular. In the context of potential expansion of postpartum Medicaid coverage from 60 days to one year, we sought to capture the societal, financial burden of untreated MMHCs.

**Methods:**

We estimated the economic impact of untreated maternal mental health conditions (MMHCs) among births in Texas in 2019 using a cost-of-illness model.

**Results:**

We found that MMHCs affected 13.2% of mothers and, when left untreated, cost $2.2 billion among mothers and children born in Texas in 2019 when following the birth cohort from conception through five years postpartum. We found that MMHCs affected 17.2% of mothers enrolled in Texas’ Medicaid for Pregnant Women and cost $962 million. In addition, the prevalence of MMHCs and resulting costs varied considerably among women of different races and ethnicities. Employers and health care payers, including Medicaid, bore most of these costs.

**Conclusions:**

The Texas Health and Human Services Commission’s (HHSC) efforts to increase awareness about MMHCs and increase access to care represent an important step toward improving maternal and child health and maximizing benefits to Texas HHSC, employers, and insurers.

**Supplementary Information:**

The online version contains supplementary material available at 10.1186/s12884-022-05001-6.

## Background

Maternal mental health conditions (MMHCs), which include depression and anxiety disorders during pregnancy and through five years postpartum, affect at least 1 in 8 pregnant and postpartum women in the U.S. and are among the most common obstetric complications in Texas [1]. Although effective screening tools and treatments exist, these conditions frequently go undiagnosed and untreated [2, 3]. In fact, only about 40% of women who receive a diagnosis obtain adequate treatment [3]. When left untreated, MMHCs may become a multigenerational issue, affecting the mother and child’s physical and emotional health and overall wellbeing. MMHCs may lead to reduced maternal economic productivity through an inability to work or adequately perform work duties, an increased risk of suicide, increased use of public services such as Medicaid, and worse maternal and child health [4].

In Texas, legislators and health insurers have paid increasing attention to MMHCs, as evidenced by the Texas Health and Human Services Commission’s (HHSC) September 2020 release of a plan to address postpartum depression [5]. To support further efforts to increase screening and expand access to effective treatment, we produced detailed estimates of the economic impact of not treating MMHCs, that is the excess burden of untreated MMHCs relative to not having MMHCs in the population.

**Fig. 1:**
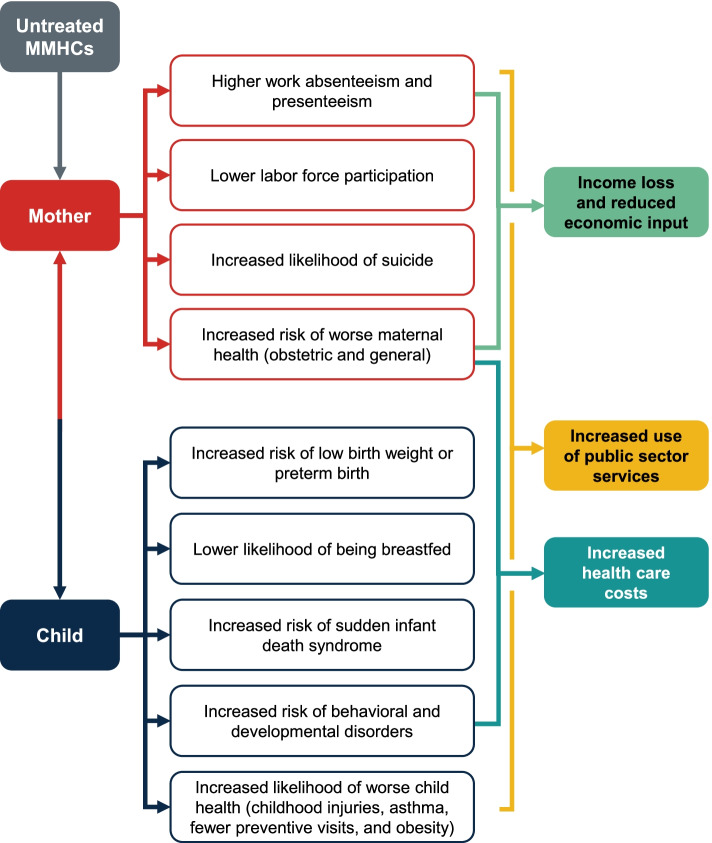
Conceptual framework: Influence of untreated MMHCs on maternal and child outcomes. SOURCE: Authors’ systematic review of the literature. NOTES: Mothers with MMHCs have a greater risk of presenteeism (reduced productivity and accuracy at work), absenteeism (regularly missing work), unemployment, and suicide. They are also more likely to experience preeclampsia or cesarean delivery, to have a long post-delivery hospital stay, and to have high non-obstetric health care costs. Children of mothers with MMHCs have a higher risk of being born preterm, not being breastfed, dying of sudden infant death syndrome, or having physical health issues. In addition, they are more likely to have a behavioral or developmental disorder, such as attention-deficit/hyperactivity disorder, depression, anxiety, and behavioral or conduct disorders such as oppositional defiant disorder, which can lead to reduced educational attainment in the longer term. MMHCs = maternal mental health conditions

In Luca et al. (2020), we developed a conceptual framework to quantify the economic impact of untreated MMHCs nationally (Fig. 1) [6]. In this study, we used the conceptual framework we developed in Luca et al. (2020), as well as recent data and estimates from peer-reviewed literature, to quantify the societal costs of untreated MMHCs in Texas. To our knowledge, this study represents the first effort to quantify the costs of untreated MMHCs in Texas, and to quantify the economic impact of untreated MMHCs by enrollment in Texas Medicaid for Pregnant Women and by race and ethnicity.

Our mathematical model accounts for costs incurred by the mother–child pair from conception through five years post-delivery to highlight the immediate associations most relevant to the public and to policymakers. Since exposure to MMHCs can also impact a child’s well-being in the longer term, our estimates should be interpreted as a lower bound on the true cost of untreated MMHCs in Texas.

## Methods

To build our cost-of-illness model, we focused on Texas-specific maternal and child outcomes linked to MMHCs in the literature and recognized by subject matter experts. We estimated societal costs incurred by mothers and their children born in 2019 and projected these costs from conception through the child’s fifth birthday. We accounted for three types of direct and indirect costs: (1) maternal income loss due to lower labor force participation and productivity losses; (2) increased use of public assistance, including Supplemental Nutrition Assistance Program (SNAP), Special Supplemental Nutrition Program for Women, Infants and Children (WIC), and Medicaid; and (3) higher health care costs due to worse maternal and child health.

We synthesized evidence on the associations between untreated MMHCs and maternal and child outcomes. We compiled data from peer-reviewed literature and secondary data sources to determine model inputs, which included: (1) the prevalence of MMHCs in Texas; (2) measures of association to measure the incremental effect of exposure to untreated MMHCs relative to no exposure; and (3) costs and baseline rates for each outcome.

### Literature review

We conducted a systematic literature search following the Preferred Reporting Items for Systematic Reviews and Meta-Analyses (PRISMA) guidelines to obtain measures of association on the relationship between exposure to untreated MMHCs and maternal and child outcomes [7]. We applied three key inclusion criteria to guide our selection of articles: (1) studies had to use adequate controls to verify that the reported effects occurred to MMHC exposure, rather than to potentially confounding factors (either by adjusting for confounding variables or by using a valid matched comparison design); (2) outcomes had to have relevance to the one-, two-, and five-year time frames of the model; and (3) the study had to quantify outcomes in monetary terms. We sought to identify credible, statistically significant measures of association for use in our model; we excluded papers reporting only statistically non-significant results. We retained papers that met quality standards and had significant results for some outcomes of interests and nonsignificant results for others. We considered studies that sufficiently adjusted for confounding variables or used a matched comparison group design rigorous enough to have generated credible measures of association and excluded estimates from studies that did not meet these design and analysis criteria. We started with the articles from Luca et al. (2020), and used the Ovid MEDLINE, CINAHL, Cochrane, APA PsycInfo, and Scopus databases and key search terms to identify newer articles published in peer-reviewed journals. To include the most current and relevant evidence, we restricted articles to those published from 2008 to 2020, prioritizing articles with Texas-specific estimates where possible. We supplemented these articles with references from other publications focusing on the costs and impacts of untreated MMHCs and grey literature.

We identified 111 relevant articles that (1) controlled for confounding variables or used a matched comparison group design, (2) focused on outcomes occurring between conception and the child’s fifth birthday, and (3) estimated associations in numeric or monetary terms. Supplementary Figure 1 and Table 1 contain more information on our literature search strategy and results, and Supplementary Table 2 focuses on the measures of association we found through the literature search. For relationships where more than one measure of association was identified, we calculated the unweighted average and used this in our model. We did not weight estimates from each study by population size.

### Baseline rates

We estimated the prevalence of MMHCs in Texas and the prevalence or incidence of each outcome in the Texas population. In selecting estimates, we prioritized recency and relevance to pregnant and postpartum women and children ages 0 to 5. For example, the baseline rates of absenteeism, presenteeism, and unemployment apply to reproductive-age women or women with young children. Supplementary Table 3 contains the MMHC prevalence estimates we used in the main model and subgroup analyses.

### Cost estimates

To calculate the total cost of MMHCs in Texas, we estimated the incremental cost of each outcome attributable to exposure to untreated MMHCs. We estimated baseline costs using peer-reviewed literature and data from the Centers for Disease Control and Prevention (CDC), Bureau of Labor Statistics (BLS), and other government agencies. We collected granular estimates of direct and indirect costs from the literature, accounting for both immediate and downstream costs, and excluded overlapping cost components to avoid overstating costs. We standardized costs to annual units and converted costs to 2019 dollars using the medical component of the consumer price index (CPI) for medical costs and the CPI for all urban consumers, less food and energy, for non-medical costs. Supplementary Table 4 provides details on incremental cost estimates, the interpretation of these estimates, and data sources.

### Modeling

Using Microsoft Excel, we collated estimates and developed a cost-of-illness model to quantify the costs of MMHCs among Texas mothers giving birth in 2019 and their children (Supplementary Table 5). For each outcome, we collected a range of measures of association from studies identified in our literature review. Next, we converted the measures of association to percentage point changes in the likelihood of each outcome given exposure to untreated MMHCs.

We then calculated the expected rate of each outcome among women with MMHCs and their children by adding together the baseline rate of each outcome and the measures of association. For example, the measure of association for cesarean sections represents the incremental risk of a woman with an MMHC needing a cesarean section compared to a woman without MMHCs. The sum of the baseline rate and this estimate represents the incremental risk of a woman with an untreated MMHC needing a cesarean section. We calculated the annual incremental cost of each outcome given exposure to untreated MMHCs by multiplying together the incremental risk of the outcome, the expected number of women with MMHCs, and the per capita cost of each outcome.

Our model assumes modifiable associations of MMHCs with key outcomes; in other words, treatment for MMHCs would eliminate the excess risk of adverse outcomes. Our model reflects potential complex causal pathways from MMHCs to adverse outcomes as represented in published literature.

We projected costs through five years postpartum by assuming that:

1. Pre-eclampsia, cesarean delivery, long peripartum hospital stay, preterm birth, sudden infant death syndrome (SIDS), and suboptimal breastfeeding are potential outcomes that occur once, in Year 0 (from conception through the first year postpartum).
2. Costs of productivity loss, suicide, non-obstetric healthcare, child injury, emergency department visits, and missed well-child care visits are incurred annually until the mother achieves remission, and then fall to $0.
3. Two-thirds of women, whether treated or untreated, achieve remission at the end of Year 0. Based on a meta-analysis by Vliegen et al. (2014), the proportion achieving remission each year is constant across years [8].
4. The association between untreated MMHCs and child behavioral and developmental disorders, asthma, and obesity remains constant regardless of whether the mother achieves remission.

We then projected costs through five years postpartum by calculating the percentage change between the December 2018 and December 2019 medical CPIs. Based on the percentage change, we assumed that medical costs increased by 4.53% annually beyond Year 0 [9]. Finally, following a recommendation by the U.S. Preventive Services Task Force, we discounted costs at an annual rate of 3%. This reflects the lower present economic value of expenses incurred in the future [10]. We summed together discounted costs to estimate the total cost of each outcome.

To understand the sensitivity of our cost estimates to variation in prevalence, cost, and measures of association, we conducted one-way deterministic sensitivity analyses, calculating differences from the main model as we varied each parameter from the lowest to highest value identified in the literature. (If only one estimate of a parameter appeared in the literature, we used the upper and lower bounds of the 95% confidence interval as the lowest and highest values).

## Results

### All Texas mothers

The total estimated cost of exposure to untreated MMHCs in Texas was $2.2 billion for the 2019 birth cohort, accounting for costs incurred in the six years between conception and five years post-delivery (Table 1). Assuming that 49,816 out of 377,397 Texas mothers (13.2%) who gave birth in 2019 had an untreated MMHC, these conditions cost more than $44,000 per affected mother–child pair over the six model years [1, 11]. This cost is higher than the national average of $32,000 that we calculated in Luca et al. (2020) Nearly half of these costs are incurred in Year 0 and are attributable to obstetric complications, pre-term birth, suboptimal breastfeeding, and SIDS.

**Table 1:** Model results for costs of untreated MMHCs (in millions of dollars) for the 2019 birth cohort: All Texas mothers

Outcomes	Total	Year 0	Year 1	Year 2	Year 3	Year 4	Year 5
**Maternal costs**							
Productivity losses	610.2	222.7	77.5	81.0	84.7	88.5	92.5
Suicide	12.0	4.4	1.5	1.6	1.7	1.7	1.8
Preeclampsia^a^	36.8	36.8	0.0	0.0	0.0	0.0	0.0
Cesarean delivery^a^	55.2	55.2	0.0	0.0	0.0	0.0	0.0
Peripartum stay^a^	31.3	31.3	0.0	0.0	0.0	0.0	0.0
Non-obstetric health expenditures	445.1	162.4	56.5	59.1	61.8	64.6	67.5
Benefit receipt	22.2	3.5	3.7	3.9	4.1	4.3	4.5
**Child costs**							
Preterm birth^a^	371.8	371.8	0.0	0.0	0.0	0.0	0.0
Suboptimal breastfeeding^a^	5.7	5.7	0.0	0.0	0.0	0.0	0.0
SIDS	1.3	1.3	0.0	0.0	0.0	0.0	0.0
Behavioral and developmental disorders	555.7	89.3	93.3	97.5	101.9	106.5	111.3
Obesity	3.9	0.7	0.7	0.7	0.7	0.7	0.7
Asthma	32.8	5.3	5.5	5.7	6.0	6.3	6.6
Injury	26.3	9.6	3.3	3.5	3.7	3.8	4.0
Emergency department visits	15.7	5.7	2.0	2.1	2.2	2.3	2.4
Non-attendance of well-child care visits	-11.2	-4.1	-1.4	-1.5	-1.6	-1.6	-1.7
Total societal costs for one birth cohort (millions of $)	**2,215**	**1,002**	**243**	**254**	**265**	**277**	**290**
Cost per mother–child pair with an MMHC in the first year post-delivery ($)	**20,106**						
Cost per mother–child pair with an MMHC in the first two years post-delivery ($)	**24,976**						
Cost per mother–child pair with an MMHC, averaged over the first two years post-delivery ($)	**12,488**						
Cost per mother–child pair with an MMHC in the first five years post-delivery ($)	**44,460**						
Cost per mother–child pair with an MMHC, averaged over the six years from conception through the first five years post-delivery ($)	**7,411**						

SOURCE: Authors’ analysis

^a^Costs only apply to Year 0, the year of conception and birth. We assumed that other costs incur annually through Year 5

*MMHCs* Maternal mental health conditions, *SIDS* Sudden infant death syndrome

Overall, 55% of costs are attributable to maternal outcomes. The highest-cost maternal outcomes over the six model years were productivity losses ($610.2 million), non-obstetric health expenditures ($445.1 million), and obstetric-specific health expenditures for excess cases of preeclampsia ($36.8 million), cesarean section ($55.2 million), and long peripartum inpatient stays ($31.3 million). The remaining 45% of costs are attributable to child outcomes. The highest-cost child outcomes over the six model years were child behavioral and developmental disorders ($556 million), preterm births ($372 million), and asthma ($33 million). Although missed well-child care visits could partially offset these costs in the short term (-$11.2 million), they are likely to lead to higher costs in the longer term due to worse child health.

### Mothers enrolled in Texas medicaid for pregnant women

The total estimated cost of exposure to untreated MMHCs among Texas Medicaid for Pregnant Women enrollees giving birth in 2019 was $962 million, accounting for costs incurred in the six years between conception and five years postpartum (Table 2). Assuming that 30,833 out of 179,264 enrolled mothers (17.2%) who gave birth in 2019 had an untreated MMHC, these conditions cost more than $31,000 per affected mother–child pair over the six model years [1, 12]. Medicaid is separate from other programs, including SNAP, WIC, and Temporary Assistance for Needy Families (TANF), and does not pay for productivity losses, suicide, or SIDS, so we set these costs to $0 in this subgroup analysis.

**Table 2:** Model results for costs of untreated MMHCs (in millions of dollars) for the 2019 birth cohort: Mothers enrolled in Texas Medicaid for Pregnant Women

Outcomes	Total	Year 0	Year 1	Year 2	Year 3	Year 4	Year 5
**Maternal costs**							
Productivity losses	0.0	0.0	0.0	0.0	0.0	0.0	0.0
Suicide	0.0	0.0	0.0	0.0	0.0	0.0	0.0
Preeclampsia^a^	23.4	23.4	0.0	0.0	0.0	0.0	0.0
Cesarean delivery^a^	14.8	14.8	0.0	0.0	0.0	0.0	0.0
Peripartum stay^a^	17.0	17.0	0.0	0.0	0.0	0.0	0.0
Non-obstetric health expenditures	275.5	100.5	35.0	36.6	38.2	40.0	41.8
Benefit receipt	9.7	1.5	1.6	1.7	1.8	1.9	2.0
**Child costs**							
Preterm birth^a^	231.4	231.4	0.0	0.0	0.0	0.0	0.0
Suboptimal breastfeeding^a^	3.6	3.6	0.0	0.0	0.0	0.0	0.0
SIDS	0.0	0.0	0.0	0.0	0.0	0.0	0.0
Behavioral and developmental disorders	344.8	55.4	57.9	60.5	63.2	66.1	69.1
Obesity	2.2	0.4	0.4	0.4	0.4	0.4	0.4
Asthma	20.6	3.3	3.4	3.6	3.8	4.0	4.2
Injury	16.1	5.9	2.1	2.1	2.2	2.3	2.5
Emergency department visits	5.3	1.9	0.7	0.7	0.7	0.8	0.8
Non-attendance of well-child care visits	-2.2	-0.8	-0.3	-0.3	-0.3	-0.3	-0.3
Total societal costs for one birth cohort (millions of $)	**962**	**458**	**101**	**105**	**110**	**115**	**121**
Cost per mother–child pair with an MMHC in the first year post-delivery ($)	**14,864**						
Cost per mother–child pair with an MMHC in the first two years post-delivery ($)	**18,133**						
Cost per mother–child pair with an MMHC, averaged over the first two years post-delivery ($)	**9,067**						
Cost per mother–child pair with an MMHC in the first five years post-delivery ($)	**31,200**						
Cost per mother–child pair with an MMHC, averaged over the six years from conception through the first five years post-delivery ($)	**5,200**						

SOURCE: Authors’ analysis

^a^Costs only apply to Year 0, the year of conception and birth. We assumed that other costs are incurred annually through Year 5

*MMHCs* Maternal mental health conditions, *SIDS* Sudden infant death syndrome

In this population, 35% of costs are attributable to maternal outcomes, with non-obstetric health expenditures ($276 million) yielding the highest cost. The remaining 65% of costs are attributable to child outcomes, with child behavioral and developmental disorders ($345 million) and preterm birth ($231 million representing the largest costs. Historically, Medicaid income eligibility limits in Texas have been low [13, 14], and coverage through Texas Medicaid for Pregnant Women has lapsed after 60 days postpartum, though Texas lawmakers are currently working to extend coverage through a full year postpartum [15]. As a result, a majority of these costs are borne by the health care system (for uninsured or underinsured women and their children) or by insurers. Supplementary Table 6 provides details on the point estimates and data sources we used for this subgroup analysis.

### Differences by race and ethnicity

We estimated costs for non-Hispanic White, non-Hispanic Black, and Hispanic mothers to examine potential differences in how MMHCs impact Texas mothers of different racial and ethnic backgrounds and their children. Although we aimed to assess potential differences among women of other backgrounds, we found that stratified data are most consistently available for these racial and ethnic groups. We assumed that MMHCs have the same impact on outcomes across racial and ethnic groups and used measures of association from the main model in our subgroup models. Differences in costs across racial and ethnic groups appeared driven primarily by differences in the prevalence of MMHCs across these groups.

We accounted for differences in population size, as well as the prevalence and societal and health outcomes of MMHCs and found that untreated MMHCs are most prevalent among non-Hispanic Black mothers (18.2%), followed by Hispanic mothers (12%) and non-Hispanic White mothers (11.4%) [1]. These health disparities lead to higher societal costs per non-Hispanic Black mother–child pair (nearly $62,000) than for Hispanic and non-Hispanic White mother–child pairs (about $43,000). Table 3 presents a breakdown of costs by racial and ethnic group, Supplementary Tables 7, 8 and 9 present more detailed results, and Supplementary Table 10 presents model inputs and data sources.

**Table 3:** Model results for costs of untreated MMHCs (in millions of dollars) for the 2019 birth cohort: Mothers by race/ethnicity group

Outcomes	Non-Hispanic White	Non-Hispanic Black	Hispanic
**Maternal costs**			
Productivity losses	169.5	189.4	229.0
Suicide	8.0	2.4	3.6
Preeclampsia^a^	8.8	8.8	16.6
Cesarean delivery^a^	14.7	9.5	22.7
Peripartum stay^a^	8.7	5.3	13.5
Non-obstetric health expenditures	122.7	117.5	211.0
Benefit receipt	5.6	5.0	10.3
**Child costs**			
Preterm birth^a^	89.2	74.0	149.2
Suboptimal breastfeeding^a^	1.6	1.0	2.5
SIDS	0.4	0.5	0.5
Behavioral and developmental disorders	154.9	93.9	238.9
Obesity	1.1	0.6	2.2
Asthma	4.5	11.4	17.5
Injury	7.1	3.3	7.1
Emergency department visits	5.3	0.8	8.1
Non-attendance of well-child care visits	-3.3	-2.0	-4.9
Total societal costs for one birth cohort (millions of $)	**599**	**521**	**928**
Cost per mother–child pair with an MMHC in the first year post-delivery ($)	**18,941**	**27,308**	**19,327**
Cost per mother–child pair with an MMHC in the first two years post-delivery ($)	**23,784**	**34,209**	**24,131**
Cost per mother–child pair with an MMHC, averaged over the first two years post-delivery ($)	**11,892**	**17,105**	**12,066**
Cost per mother–child pair with an MMHC in the first five years post-delivery ($)	**43,106**	**61,671**	**43,322**
Cost per mother–child pair with an MMHC, averaged over the six years from conception through the first five years post-delivery ($)	**7,184**	**10,279**	**7,220**

SOURCE: Authors’ analysis

Please refer to Supplementary Tables 7, 8 and 9 for annual costs for non-Hispanic White, non-Hispanic Black, and Hispanic mother–child pairs

The total estimated cost of untreated MMHCs among non-Hispanic White mother–child pairs was $599 million for the 2019 birth cohort, accounting for costs incurred over six model years. Assuming that 13,896 of the 121,899 Non-Hispanic White mothers (11.4%) who gave birth in 2019 had an untreated MMHC [1], these conditions cost $43,106 per affected non-Hispanic White mother–child pair over six years. In this population, 56% of costs are attributable to maternal outcomes, with productivity losses ($170 million) and non-obstetric health expenditures ($123 million) yielding the highest costs. The remaining 44% of costs relate to child outcomes, with child behavioral and developmental disorders ($155 million) and preterm birth ($89 million) yielding the highest costs.

The total estimated cost of exposure to untreated MMHCs among non-Hispanic Black mother–child pairs was $521 million. Assuming 8,448 of the 46,420 non-Hispanic Black mothers (18.2%) who gave birth in 2019 had an untreated MMHC, these conditions cost $61,671 per affected non-Hispanic Black mother–child pair over six years. In this population, 65% of these costs relate to maternal outcomes, with productivity losses ($189 million) and non-obstetric health expenditures ($118 million) yielding the highest costs. The remaining 35% of costs relate to child outcomes, with child behavioral and developmental disorders ($94 million) and preterm birth ($74 million) yielding the highest costs.

Finally, the total estimated cost of exposure to untreated MMHCs among Hispanic mother–child pairs was $928 million. Assuming 21,421 of the 178,509 Hispanic mothers (12%) who gave birth in 2019 had an untreated MMHC, these conditions cost $43,322 per affected Hispanic mother–child pair over six years. In this population, 55% of these costs relate to maternal outcomes, with productivity losses ($229 million) and non-obstetric health expenditures ($211 million) yielding the highest costs. The remaining 45% of costs relate to child outcomes, with child behavioral and developmental disorders ($239 million) and preterm birth ($149 million) yielding the highest costs.

The magnitude of the disparities in the prevalence and cost of untreated MMHCs suggests ample room for policy interventions to ensure more equitable access to screening and effective treatment options. In the long run, these interventions could reduce suffering caused by untreated MMHCs as well as reduce costs to employers, the health care system, insurers, and the government.

### Sensitivity analysis

**Fig. 2:**
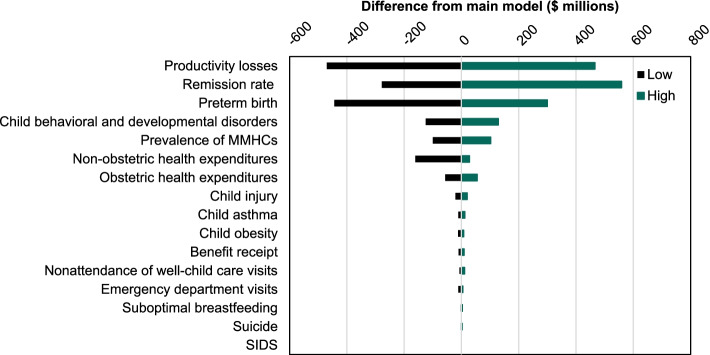
Tornado diagram from one-way sensitivity analyses of the economic impact of untreated MMHCs among 2019 births: All Texas mothers. SOURCE: Authors’ analysis. NOTES: MMHCs = maternal mental health conditions; SIDS = sudden infant death syndrome. The tornado diagram above shows the results of our sensitivity analyses, including the difference in costs, in millions of dollars, from the main model as we varied each parameter from its lowest to its highest value. The impact estimate of exposure to MMHCs on productivity losses had the greatest impact on estimated costs ($1.7 billion to $2.7 billion), followed by the remission rate ($1.9 billion to $2.8 billion) and preterm birth ($1.8 billion to $2.3 billion). These parameters had large ranges in the literature and the costs of these outcomes were very high, so varying these parameters led to substantial variation in model results. Other impact parameters had a more modest impact on model results because the baseline rate of the outcome was low, the costs incurred through five years post-delivery were low or the range of impact estimates was small. By varying all parameters at once, we found that total societal cost of untreated MMHCs in Texas could range from $739 million to $4.3 billion for the 2019 birth cohort

The sensitivity analysis showed that our model estimated a range of $739 million to $4.3 billion for the 2019 Texas birth cohort through varying all prevalence and impact estimates at once (Fig. 2). The measure of association between MMHCs and productivity losses had the greatest impact on estimated costs ($1.7 billion to $2.7 billion), followed by the MMHC remission rate ($1.9 billion to $2.8 billion) and preterm birth ($1.8 billion to $2.3 billion). Parameters with large ranges in the literature and the costs of these outcomes meant that varying these parameters led to substantial variation in model results.

Other parameters had a more modest impact on model results because of a low baseline rate of the outcome, low costs incurred through five years post-delivery, or narrow range of measures of association.

### Limitations

This study had several limitations. First, our literature review focused on identifying statistically significant estimates for use in our model. Since we did not use estimates that did not reach statistical significance, our model may exclude relevant and important relationships between MMHCs and maternal or child outcomes for which statistically significant associations have not appeared in the literature; publication bias may exacerbate this issue (e.g., nonsignificant results may have a lower likelihood of appearing in the literature than significant findings). Although we focused on immediate associations in the six-year period between conception and five years post-delivery, we recognize that MMHCs can have long-term impacts on mothers and their children. For example, children who develop behavioral and developmental disorders due to exposure to MMHCs could have lower educational attainment. Our model does not account for the increased prevalence of MMHCs arising from the COVID-19 pandemic. We did not account for the impact of MMHCs on nonmaternal caregivers, though we recognize they may also have mental health conditions. In addition, most of the literature on the impacts of untreated MMHCs focused on maternal depression rather than maternal anxiety. If maternal anxiety impacts outcomes independently of maternal depression and a mother has both anxiety and depression, the model may underestimate costs of untreated MMHCs [16]. As a result, our model results should be interpreted as conservative estimates of the true cost. Similarly, our model assumes independent associations between MMHCs and various maternal and child outcomes. Although we conducted a sensitivity analysis to understand the possible range of cost estimates we could have generated based on our inputs, there may still be some imprecision in our estimates. For example, MMHCs remain under-detected, which could contribute to an underestimate in the overall model.

Additionally, we did not account for treatment options and did not distinguish between untreated and inadequately treated MMHCs, so did not make the economic case for treatment, or estimate potential cost savings through treatment of MMHCs. Finally, we did not analyze primary data, but rather used inputs from the literature and secondary data sources.

## Discussion

To inform financial and policy approaches to improving screening for and treatment of MMHCs in Texas, we estimated the total societal cost of untreated MMHCs, as well as costs among mothers enrolled in Texas Medicaid for Pregnant Women and their children. To uncover potential disparities in disease burden, we also quantified costs among non-Hispanic White, non-Hispanic Black, and Hispanic mother–child pairs.

We found that untreated MMHCs generated excess costs of $2.2 billion relative to cases in which MMHCs were not experienced among Texas mothers and children in the 2019 birth cohort. This amounts to more than $44,000 per mother–child pair from conception through the child’s fifth birthday. Nearly half of the total costs are incurred before the child’s first birthday, and overall, 55% of costs are attributable to maternal outcomes and 45% of costs are attributable to child outcomes.

Among births covered by Texas Medicaid for Pregnant Women, we found that the total cost of exposure to untreated MMHCs was $962 million, or $31,071 per pair, excluding costs of productivity loss, maternal suicide, and SIDS. Maternal outcomes, especially non-obstetric health issues ($276 million), accounted for 35% of total costs. Child outcomes, especially child behavioral and developmental disorders ($345 million) and preterm birth ($231 million), accounted for 65% of costs.

Given termination of coverage through this program after 60 days postpartum, low income eligibility requirements, and the fact that 57% of costs are incurred beyond the birth year, Medicaid directly bears only a small portion of these costs. If a mother cannot afford to purchase health insurance and does not meet Medicaid income eligibility requirements, the health care system could bear the costs of adverse health outcomes for mother and child. If a mother can afford to purchase insurance through the Affordable Care Act Marketplace, insurers may bear these costs.

We also found evidence of substantial disparities in the disease burden of untreated MMHCs among mothers of different racial and ethnic backgrounds. From conception through five years postpartum, untreated MMHCs cost $599 million among non-Hispanic White mother–child pairs ($43,106 per pair), $521 million among non-Hispanic Black mother–child pairs ($61,671 per pair), and $928 million among Hispanic mothers ($43,322 per pair). The distribution of costs for non-Hispanic White and Hispanic mother–child pairs was comparable to the distribution among the general population, but for non-Hispanic Black mother–child pairs, the distribution was more heavily skewed toward maternal outcomes (65% of total costs, with 35% of total costs pertaining to child outcomes).

## Conclusions

Our study shows that failing to treat MMHCs imposes a heavy cost on society ($2.2 billion) in Texas. It also shows that employers, through reduced employee productivity, and health insurers, through worse maternal and child health, bear most of these costs.

We also found substantial variation in both the prevalence and cost of MMHCs among mother–child pairs of different racial and ethnic backgrounds. These disparities in disease burden may be attributable to poorer access to screening and high-quality care among women impacted by systemic racism and socioeconomic disadvantage and highlight a need for targeted interventions. Improving access to screening and treatment for MMHCs among women of all races and ethnicities would help increase women’s productivity, reduce the need for high-cost maternal and pediatric health care, and decrease the need for public assistance. This would lead to lower costs to Texas HHSC, employers, insurers, and the health care system, and would help support the health of Texas mothers and their children for generations to come.

## Supplementary Information

**Additional file 1: Supplementary Figure 1.** Literature review: PRISMA flowchart of article selection. **Supplementary Table 1.** Literature Review: search terms. **Supplementary Table 2.** Effects of exposure to MMHCs. **Supplementary Table 3.** Prevalence of MMHCs. **Supplementary Table 4.** Studies and data sources used to inform the cost estimates used in the main model. **Supplementary Table 5.** Model Inputs (main model): Parameters and costs used to estimate the economic impact of untreated MMHCs among 2019 births. **Supplementary Table 6.** Model inputs (women enrolled in Texas Medicaid for Pregnant Women): Parameters and costs used to estimate the economic impact of untreated MMHCs among 2019 births. **Supplementary Table 7.** Model results for costs of untreated MMHCs (in millions of dollars) for the 2019 birth cohort: Non-Hispanic White mothers. **Supplementary Table 8.** Model results for costs of untreated MMHCs (in millions of dollars) for the 2019 birth cohort: Non-Hispanic Black mothers. **Supplementary Table 9.** Model results for costs of untreated MMHCs (in millions of dollars) for the 2019 birth cohort: Hispanic mothers. **Supplementary Table 10.** Model inputs (Subgroup analysis by maternal race and ethnicity): Parameters and costs used to estimate the economic impact of untreated MMHCs among 2019 Births.

## Data Availability

All data generated or analyzed during this study appear in this published article and its supplementary files.

## Abbreviations

BLS: Bureau of Labor Statistics
CDC: Centers for Disease Control and Prevention
CPI: Consumer Price Index
HHSC: Health and Human Services Commission
MMHCs: Maternal Mental Health Conditions
PRISMA: Preferred Reporting Items for Systematic Reviews and Meta-Analyses
SIDS: Sudden Infant Death Syndrome
SNAP: Supplemental Nutrition Assistance Program
WIC: Special Supplemental Nutrition Program for Women, Infants, and Children
TANF: Temporary Assistance for Needy Families
