# Supplementary material for: The economic impact of untreated maternal mental health conditions in Texas
Source: BMC Pregnancy Childbirth. 2022 Sep 12;22:700. doi: 10.1186/s12884-022-05001-6 (PMC9464607; doi:10.1186/s12884-022-05001-6)
Supplement: Supplementary file 1 — Additional file 1: Supplementary Figure 1. Literature review: PRISMA flowchart of article selection. Supplementary Table 1. Literature Review: search terms. Supplementary Table 2. Effects of exposure to MMHCs. Supplementary Table 3. Prevalence of MMHCs. Supplementary Table 4. Studies and data sources used to inform the cost estimates used in the main model. Supplementary Table 5. Model Inputs (main model): Parameters and costs used to estimate the economic impact of untreated MMHCs among 2019 births. Supplementary Table 6. Model inputs (women enrolled in Texas Medicaid for Pregnant Women): Parameters and costs used to estimate the economic impact of untreated MMHCs among 2019 births. Supplementary Table 7. Model results for costs of untreated MMHCs (in millions of dollars) for the 2019 birth cohort: Non-Hispanic White mothers. Supplementary Table 8. Model results for costs of untreated MMHCs (in millions of dollars) for the 2019 birth cohort: Non-Hispanic Black mothers. Supplementary Table 9. Model results for costs of untreated MMHCs (in millions of dollars) for the 2019 birth cohort: Hispanic mothers. Supplementary Table 10. Model inputs (Subgroup analysis by maternal race and ethnicity): Parameters and costs used to estimate the economic impact of untreated MMHCs among 2019 Births. [file 12884_2022_5001_MOESM1_ESM.docx]

**Supplementary File. The Economic Impact of**

**Untreated Maternal Mental Health Conditions in Texas**

OVERVIEW

This supplementary file consists of five parts:

- Supplementary Figure 1 and Table 1 detail article selection and search terms used in the literature review
- Supplementary Tables 2-5 detail sources of inputs to the main model
- Supplementary Table 6 details sources of inputs to the Texas Medicaid for Pregnant Women model
- Supplementary Tables 7-9 detail annual cost results by racial/ethnic background
- Supplementary Table 10 details sources of inputs to the subgroup models by maternal race and ethnicity racial/ethnic background

Supplementary Figure 1

Caption: Literature Review: PRISMA flowchart of article selection


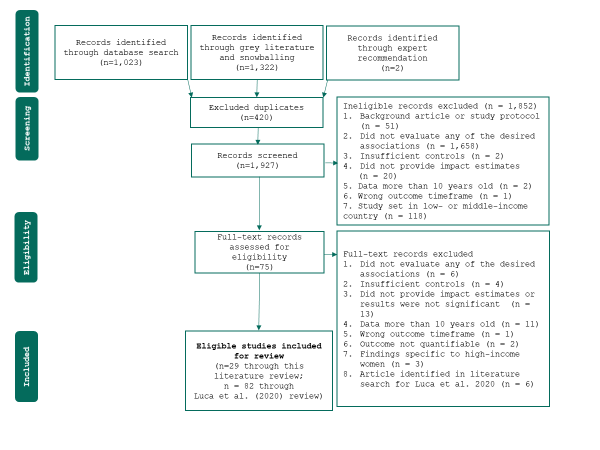


**Sources/notes:**

SOURCE: Authors’ systematic review of the literature.

Supplementary Table 1

Caption: Literature Review: search terms

| **Outcomes** | **Search terms** |
| --- | --- |
| Maternal outcomes | |
| Work | absentee*  employ*  productivity  work |
| Health | caesarean  “emergency care”  “emergency department”  “health care cost*”  “health care expenditure*”  “health services utilization"  hospitalization*  “length of stay”  “maternal health”  pre-eclampsia  “primary care”  psychiatric  psychosis  readmission* |
| Benefit Receipt | “Medicaid”  “Temporary Assistance of Needy Families”  “TANF”  “Supplemental Assistance Nutrition Program”  “SNAP”  “Special Supplemental Nutrition Program for Women Infants and Children”  “WIC” |
| Suicide | “self harm”  “self-injurious behavior”  suicid* |
| Child outcomes | |
| Health | asthma  “attention deficit/hyperactivity disorder”  autism-spectrum-disorder  behavioral-problem*  breastfeeding  child* n/3 health  conduct-problem*  conduct-disorder*  depression-or-anxiety-problem”  developmental-delay*  “emergency care”  “emergency department”  “emergency hospital”  feeding  growth  “health care cost*”  “health care expenditure*”  “health services utilization”  height  hospitalization*  immunization*  infant n/3 health  intellectual-disabilit*  learning-disabilit*  “neonatal intensive care”  “otitis media”  “preterm birth”  “preventive care”  “preventive visit*”  “primary care”  readmission*  “regular checkup*”  size  speech-problem*  “sudden infant death”  Tourette-syndrome  underweight  vaccination*  weight  “well baby care”  “well baby visit*”  “well care”  “well child care”  “wellbaby care”  “wellbaby visit*”  “wellchild care” |
| Maltreatment/neglect | child* n/3 abuse  “corporal punishment”  maltreatment  mistreatment  neglect |
| Low birthweight/preterm birth | “fetal distress”  “fetal growth”  “low birthweight”  “premature birth”  “premature delivery”  “preterm birth”  “preterm birth”  “preterm delivery”  “preterm delivery” |
| Child development | “infant development” |

**Sources/notes:**

SOURCE: Authors’ decision after consultation with in-house search experts.

NOTES: We used these search terms, along with the search terms for MMHCs, including the following: “Affective Disorders,” “Anxiety Disorders,” “Post-delivery Depression,” “Major Depression,” and “Post-Traumatic Stress” along with the subject headings for “Pregnancy” or “Mothers.” In databases that do not use subject headings, we used keywords such as “pregnant,” “antenatal,” “perinatal,” “prenatal,” “prepartum,” “post-delivery,” “maternal,” and “mother,” along with keywords for mood and anxiety disorders, such as “depression,” “depressive,” “post-traumatic stress,” “PTSD,” “anxiety,” and “mood disorder.”

* is a truncation search feature that enables users to search for any words that begin with those letters.

“n/3” is a proximity search term that means “within three words of.”

Supplementary Table 2

**Caption:** Effects of Exposure to MMHCs

| **Parameter** | **Measure of MMHCs** | **Study population** | **Sample years** | **Sample size** | **Estimate and comments** | **Methods** | **p<0.05** | **Source** |
| --- | --- | --- | --- | --- | --- | --- | --- | --- |
| Maternal outcomes | | | | | | | | |
| Remission from MMHC without treatment | Post-delivery depression | Multiple (Review) | Studies published from 1985 to 2012 | 23 studies in the final review | Around 30% of mothers with post-delivery depression continued to have major depression during their child’s first year of life, absent treatment. | Systematic review of screened longitudinal studies that have examined the course of post-delivery depression following PRISMA guidelines. We used 30% for the main model and the range of estimates (20–60%) from the review to inform the sensitivity analyses. | Y* | 1 |
| Absenteeism | Clinically diagnosed depression (self-reported) | Workers in 8 countries, including the US | 2012 | n = 1,000 per country | Annual cost of absenteeism per worker with depression is $410 (adjusted to 2019 $). | Secondary analysis on data collected in the Global IDEA (Impact of Depression in the Workplace in Europe Audit) survey to examine the effects of depression on presenteeism and absenteeism across 8 countries, controlling for country-specific contextual factors and other factors associated with the outcomes. We used the US-specific estimates to inform the model. | Y | 2 |
| Absenteeism | Major depressive disorder | Low-income mothers ages 18–35 and being unmarried, receiving Medicaid, or having incomes less than 300% of the Federal Poverty Level | 2006–2011 | n = 20,531 | Average annual cost of absenteeism per mother with depression is $619 (adjusted to 2019 $). | Analysis of the likelihood of employment and workdays missed due to major depressive disorder among mothers using data from the Medical Expenditure Panel Survey, using logistic models controlling for comorbidities, demographics, region, and year. Although their focus was on low-income mothers, they also projected cost estimates to the aggregate population, which are the estimates we use in our model. | Y | 3 |
| Absenteeism | Major depression (measured as reporting 5 or more of the 9 Diagnostic and Statistical Manual–III revised criteria for major depression in the past 2 weeks) | Depressed patients | 1996–1997 | n = 479 | Average annual cost of absenteeism per mother with depression is $899 (adjusted to 2019 $). | Randomized trial examining the impact of improved primary care depression management on absenteeism and presenteeism. A total of 479 patients were recruited from 12 community primary care practices across the US, and depression was measured as reporting 5 or more of the 9 Diagnostic and Statistical Manual–III revised criteria for major depression in the past 2 weeks. Absenteeism was measured as the total number of work hours lost due to illness or doctor visits over the past 4 weeks. | Y | 4 |
| Absenteeism | Major depressive disorder (DSM-IV criteria and ICD-9 codes) | Individuals ages 16–64 | 2005 and 2010 | n = 1,461,640 | Average annual cost of absenteeism per person with depression is $1,792 (adjusted to 2019 $). | Case-control study where individuals were matched 1-1 with controls using propensity score matching methods and national survey and administrative claims data from 2005 and 2010 to estimate the incremental burden of individuals with major depressive disorder. We used the 2010 estimates to inform the model. | Y | 5 |
| Presenteeism | Depression | Workers in 8 countries, including the US | 2012 | n = 1,000 per country | Annual cost of absenteeism per worker with depression is $410 (adjusted to 2019 $). | Secondary analysis on data collected in the Global IDEA (Impact of Depression in the Workplace in Europe Audit) survey to examine the effects of depression on presenteeism and absenteeism across 8 countries, controlling for country-specific contextual factors and other factors associated with the outcomes. We used the US-specific estimates to inform the model. | Y | 2 |
| Presenteeism | Major depression (measured as reporting 5 or more of the 9 Diagnostic and Statistical Manual–III revised criteria for major depression in the past 2 weeks) | Depressed patients | 1996–1997 | n = 479 | Average annual cost of presenteeism per mother with depression is $2,879 (adjusted to 2019 $). | Randomized trial examining the impact of improved primary care depression management on absenteeism and presenteeism. 479 patients were recruited from 12 community primary care practices across the US, and depression was measured as reporting 5 or more of the 9 Diagnostic and Statistical Manual–III revised criteria for major depression in the past 2 weeks. Productivity was self-rated by employee as “productivity as effectiveness at work over the past 2 weeks” on a scale of 0 (nothing at all accomplished) to 10 (best possible work performance). | Y | 4 |
| Presenteeism | Major depressive disorder (DSM-IV criteria and ICD-9 codes) | Individuals ages 16–64 | 2005 and 2010 | n = 1,461,640 | Average annual cost of presenteeism per person with depression is $5,804 (adjusted to 2019 $). | Case-control study where individuals were matched 1-1 with controls using propensity score matching methods and national survey and administrative claims data from 2005 and 2010 to estimate the incremental burden of individuals with major depressive disorder. We used the 2010 estimates to inform the model. | Y | 5 |
| Unemployment | Depression | Multiple (Review) | Studies published from 2002 to 2007 | Number of studies not specified | Individuals with depression have a 20–40% greater likelihood of unemployment. We used the mean (30%) for the main model and the range for the sensitivity analyses. | A review of population-based, workplace, and clinical articles reporting on the magnitude and/or nature of depression’s impact on work. | Y* | 6 |
| Suicide | Unipolar depression (hospital diagnoses) | Sweden psychiatric inpatient sample | 1973–1995 | n = 15,829 males and 23,353 females | The standardized mortality ratio for suicide for females with unipolar disorder was estimated to be 27.0. | Standardized mortality ratios by 5-year interval age at admission and time of follow-up were calculated using Poisson regression methods, controlling for calendar time of the first admission. | Y | 7 |
| Preeclampsia | Antenatal anxiety | Multiple (Review) | Studies published from 1979 to 2011 | 4 studies in the final review | OR = 3.30 (0.56 –19.37) | Systematic review of screened longitudinal studies that have examined associations with antenatal anxiety following PRISMA guidelines. DerSimonian and Laird random effects model were used to pool estimates of the odds ratios for binary outcomes and the weighted mean difference for continuous outcomes. | N | 8 |
| Preeclampsia | Maternal serious mental illness | Nationwide Inpatient Sample | 2008-2014 | n = 5,518,766 | Adjusted RR = 1.24 (1.21–1.28) | Multivariable logistic regressions adjusted for covariates: race, maternal age, insurance coverage (private insurance, Medicaid, or other), urban/rural hospital location, U.S. region, weekend, emergency department or elective admission, income, and admission year and other SMI diagnosis (MDD, BD, or schizophrenia). | Y | 9 |
| Preeclampsia | Perinatal mood and anxiety disorders | Nationwide Inpatient Sample | 2006-2015 | n = 7,906,820 | The incidence of preterm birth was higher among women with perinatal mood and anxiety disorders (9.7 per 100 deliveries) than without (6.7 per 100 deliveries). | Adjusted multivariable logistic regression models estimated delivery-related outcomes with and without perinatal mood and anxiety disorders. Models included covariate adjustments for maternal age, payer, ZIP code income quartile, rural residence, and hospital region. | Y | 10 |
| Preeclampsia | Depression (clinically diagnosed based on ICD-9 codes) at time of delivery | Nationwide Inpatient Sample | 1998–2005 | n = 3,215,6438 | OR = 1.57 (1.52–1.62) | Multivariate regression analysis examining delivery-related hospitalizations for select maternal and fetal outcomes by depression diagnosis, adjusting for age, insurance status, and hospital characteristics. | Y | 11 |
| Preeclampsia | Maternal mood and anxiety disorders diagnosed during first 20 weeks of pregnancy (self-reported and from clinical records) | Study sample recruited from women attending prenatal clinics at two hospitals in Seattle and Tacoma, WA | 1996–2004 | n = 2,601 | Adjusted RR = 3.64 (1.13–11.68) | Generalized linear regression models were used to derive relative risk (RR) estimates, controlling for maternal age, maternal race/ethnicity, and pre-pregnancy body mass index. (Other confounders were evaluated and were excluded if they did not significantly alter model coefficients.) | Y | 12 |
| Cesarean delivery | Maternal anxiety | Mother-newborn pairs with deliveries in the  Beaumont Health System. | 2013-2014 | n = 15,492 | Adjusted OR = 1.59 (1.04 –2.42) | Multinomial logistic regression compared each psychiatric diagnosis group to the unaffected referent pregnancies to calculate adjusted odds ratios controlling for baseline differences. | Y | 13 |
| Cesarean delivery | Depression | Nationwide Inpatient Sample | 1998–2005 | n = 3,215,6438 | OR = 1.34 (1.30–1.37) | Multivariable regression analysis examining delivery-related hospitalizations for select maternal and fetal outcomes by depression diagnosis. | Y | 11 |
| Cesarean delivery | Anxiety and depression (state anxiety scores >= 40 on the State Trait Anxiety Inventory (STAI) and depression scores >= 12 on the Edinburgh Postnatal Depression Survey (EPDS) were considered positive) | Mother-newborn pairs with deliveries at the Penn State Milton S. Hershey Medical Center in Hershey, PA | 2006–2009 | n = 1,154 | OR = 1.46 (1.02–2.09) | Associations of positive anxiety and depression screens at baseline with each other, demographic, maternity nursery stay–related variables, and health care use were assessed by using Chi-squared tests. A multivariable regression model was built to determine independent association with a positive anxiety screen at baseline. The relationship between anxiety and depression screen findings at baseline with breastfeeding duration was analyzed by using Kaplan-Meier methods | Y | 14 |
| Peripartum stay | Elevated CES-D (>= 16 on the CES-D) | Pregnant women recruited from obstetrics clinics in Michigan | 1999–2003 | n = 867 | An elevated CES-D was associated with a longer peripartum stay of 0.26 (0.04–0.48) days. | Multivariable Poisson regression models was used to evaluate predictors of length of stay, adjusting for sociodemographic, antepartum, and obstetric factors. | Y | 15 |
| Health expenditures | Major depressive order | Low-income mothers between ages 18–35 and being unmarried, receiving Medicaid, or having incomes less than 300% of the Federal Poverty Level | 2006–2011 | n = 20,531 | Average incremental out-of-pocket health expenditures due to depression are $340 and average incremental insurer expenditures due to depression are $1,727 (in 2019 $) | Analysis of the likelihood of employment and work days missed due to major depressive disorder among mothers using data from the Medical Expenditure Panel Survey, using logistic models controlling for comorbidities, demographics, region, and year. Although their focus was on low-income mothers, they also projected cost estimates to the aggregate population, which are the estimates we use in our model. | Y | 3 |
| SNAP receipt | Maternal depression (CES-D >= 16 assessed at 9 months post-delivery) | Early Childhood Longitudinal Survey | 2001–2003 | n = 7,900 | Probit coefficient = 0.23 (0.14–0.32) | Analysis of effects of maternal depression (assessed when the child was 9 months old) on benefit receipt (measured when the child is 2), using multivariate probit models, controlling for age, race/ethnicity, educational attainment, prenatal health, and family history of depression. | Y | 16 |
| WIC receipt | Maternal depression (assessed at 9 months post-delivery) | Early Childhood Longitudinal Survey | 2001–2003 | n = 7,900 | Probit coefficient = 0.18 (0.09–0.26) | Analysis of effects of maternal depression (assessed when the child was 9 months old) on benefit receipt (measured when the child is 2), using multivariate probit models, controlling for age, race/ethnicity, educational attainment, prenatal health, and family history of depression. | Y | 16 |
| Medicaid receipt | Maternal depression (assessed at 9 months post-delivery) | Early Childhood Longitudinal Survey | 2001–2003 | n = 7,900 | Probit coefficient = 0.24 (0.15–0.34) | Analysis of effects of maternal depression (assessed when the child was 9 months old) on benefit receipt (measured when the child is 2), using multivariate probit models, controlling for age, race/ethnicity, educational attainment, prenatal health, and family history of depression. | Y | 16 |
| TANF receipt | Maternal depression (assessed at 9 months post-delivery) | Early Childhood Longitudinal Survey | 2001–2003 | n = 7,900 | Probit coefficient = 0.20 (0.09–0.31) | Analysis of effects of maternal depression (assessed when the child was 9 months old) on benefit receipt (measured when the child is 2), using multivariate probit models, controlling for age, race/ethnicity, educational attainment, prenatal health, and family history of depression. | Y | 16 |
| Child outcomes | | | | | | | | |
| Preterm birth | Perinatal depression | Population‐  based administrative data holdings in Alberta, Canada, covering > 99.0% of the general province population | 2012-2015 | n = 158,486 | Adjusted RR: 1.49 – (1.411.58) | Multivariable log‐binomial regression models were used to assess the risk of adverse outcomes associated  with depression alone (compared to without depression), adjusting for age and parity. | Y | 17 |
| Preterm birth | Maternal serious mental illness | Nationwide Inpatient Sample | 2008-2014 | n =5,518,766 | Adjusted RR = 1.19 (1.16–1.22) | Multivariable logistic regressions adjusted for covariates: race, maternal age, insurance coverage (private insurance, Medicaid, or other), urban/rural hospital location, U.S. region, weekend, emergency department or elective admission, income, and admission year and other SMI diagnosis (MDD, BD, or schizophrenia). | Y | 9 |
| Preterm birth | Depression assessed using either a clinical interview/diagnosis or a screening tool or scale at any time during pregnancy | Varies (review) | No publication date restriction | 23 studies included in the final review | Among high-quality US studies, ORs for preterm birth ranged from OR = 0.71 (0.47–1.07) to OR = 4.97 (1.54–16.05) | Systematic review examining randomized and nonrandomized studies reporting the risk of adverse neonatal outcomes in pregnant women with untreated depression compared with pregnant women without depression. | Y* | 18 |
| Suboptimal breastfeeding | Post-delivery depression and anxiety (self-reported) | Pregnancy Risk Assessment Monitoring System | 2010–2011 | n = 55,987 | Any breastfeeding at 3 months: OR = 0.79 (0.70–0.88) Exclusive breastfeeding at 3 months: OR = 0.58 (0.50–0.68) Anxiety: OR = 0.87 (0.70–1.08) (any breastfeeding) OR = 0.92 (0.68–1.24) exclusive | Multivariable logistic regression was used to explore the association between a pre-pregnancy mental health visit and subsequent breastfeeding initiation as well as PPD and 3-month any and exclusive breastfeeding, controlling for adjusted for maternal race/ethnicity, age, marital status, pre-pregnancy mental health visit, and prenatal morbidity, abuse during or in the 12 months before pregnancy, and delivery type. | Y | 19 |
| SIDS | Antenatal (a year before delivery) and postnatal depression (6 months post-delivery) (clinical diagnoses ICD-10 codes F32–F38) | Female patients registered in the UK General Practice Research Database | 1987–2000 | n = 169 cases; n = 662 controls | OR = 4.93 (1.10–22.05) | Case-control study of women with a live birth and subsequent SIDS death, compared to women with a live birth born the same year as the matched SIDS death, with infant survival for the first year of life. | Y | 20 |
| SIDS | Depression (EPDS >= 12 at 1 month post-delivery) | All births registered in Sheffield, UK | 1988–1993 | n = 32,984 | OR = 3.20 (1.46–6.99) | Case-control study comparing the rate of SIDS among mothers who had a high EPDS score versus those who did not, controlling for mothers’ smoking status, residence in an area of poverty, preterm birth, maternal age, number of previous pregnancies, birth weight, number born (multiple births), maternal psychiatric history, year of birth (within study), month or season of birth, number of health visitor visits, baby’s sex, mother’s feeding intention (breast or bottle), mode of feeding at 1 month (breast or bottle), or maternal satisfaction with the infant’s feeding. | Y | 21 |
| Any behavioral and developmental disorders based on total SDQ scores | Elevated prenatal depression and anxiety at 32 weeks gestation (top 15%) (Crown–Crisp Experiential Index) (EPDS >= 13) | Pregnant women residing in the Avon area of southwest England | Women who had an estimated date of delivery between April 1, 1991, and December 31, 1992 | n = 7,944 | Probable child mental disorder: OR = 1.8 (1.62–1.98) | Longitudinal cohort study showing that maternal prenatal anxiety and depression at 32 weeks predicted greater child emotional and behavioral problems independent of a range of confounders (maternal age and education, crowding as index of socioeconomic status, birth weight and gestational age of the child, child sex, maternal prenatal smoking and substance use, maternal postnatal depression and anxiety, paternal pre- and postnatal anxiety, and a parenting index). | Y | 22 |
| Child obesity | Maternal depression | Fragile Families and Child Wellbeing Study | 2009 | n = 2,965 | Adjusted OR: 1.35 (1.06–1.72) | Stepped multivariate analyses estimated the relationships between child obesity and maternal depression, adjusting for sociodemographic characteristics, child health and health behaviors, maternal health factors, and maternal–child relationship factors. | Y | 23 |
| Child obesity | Perinatal depression (CESD≥16 or EPDS≥13) | Latina mothers and their infants from 2 medical centers in San Francisco, CA (sample excluded women with drugs or alcohol abuse, diabetes, polycystic ovarian syndrome, eating disorders, or any health problems that would affect  breastfeeding.) | 2006–2007 | n = 181 | Decreased chance of overweight (OR = 0.28, 95%; CI = 0.03 –0.92) | Longitudinal cohort study examining association between exposure to perinatal maternal depression and child weight-for-length z-score at 6,12, and 24 months, controlling for infant birth weight, breastfeeding status, maternal postnatal BMI, maternal ethnicity, maternal age and gestational age. | Y | 24 |
| Child obesity | Maternal anxiety and depression | Various (Review) | Studies published between 2000 and 2014 | 2,033 records identified and 20 studies included in final review | OR ranges from 0.28 (0.03–0.92) to 2.62 (1.02–6.70) | Systematic review. Majority of studies showed positive associations between maternal depressive symptoms and increased risks for preschooler obesity. Effect sizes varied depending on the time at which depression was measured (i.e., antenatal, postnatal, in isolation, or longitudinally). | Y* | 25 |
| Asthma | Elevated prenatal depression and anxiety at 32 weeks gestation (top 15%); anxiety was assessed using anxiety subscale of the Crown-Crisp Experiential Index. Depression was assessed using the EPDS | Pregnant women residing in the Avon area of southwest England | 1991–1999 | n = 5,810 | OR = 1.64 (1.25–2.17) | Longitudinal cohort study showing that maternal prenatal anxiety and depression at 32 weeks predicted greater child emotional and behavioral problems independent of a range of confounders (maternal age and education, crowding as index of socioeconomic status, birth weight and gestational age of the child, child sex, maternal prenatal smoking and substance use, maternal postnatal depression and anxiety, paternal pre- and postnatal anxiety, and a parenting index). | Y | 26 |
| Asthma | Depression (Kessler-6 >= 13) | Nationally representative cohort of Australian children | 2004–2011 | n = 4,164 | OR = 2.36 (1.61–3.45) | Longitudinal cohort study using logistic regression analyses, controlling for risk factors, including child gender, maternal smoking during pregnancy, maternal use of asthma medication during pregnancy, instrumental delivery (cesarean, vacuum extraction, and/or forceps), preterm birth (< 37 weeks), low birth weight (< 2,500 grams), not being breastfed, attending a child care center within the first year of life, maternal age, number of children in the family, living in a metropolitan area, and socioeconomic status. | Y | 27 |
| Child injury | Depression (CESD >= 16) | Study of Early Child Care (sample from 10 US cities) | Not given | n = 1,364 | Beta = 1.06 (0.58, 1.54) | Use Poisson models to assess maternal depression's effect on child injuries from birth to age 3, controlling for family socioeconomic background, child sex, child temperament and externalizing behavior, and parenting behaviors. | Y | 28 |
| Child injury | Depression (EPDS >= 9) | Japanese mothers/children | 2012 | n = 9,707 | OR = 1.59 (1.24–2.04) | Cross-sectional study using logistic multivariate regression to analyze the association between post-delivery depression and experience of any unintentional injury, falls, and near drowning of infant children (4 months), adjusted for maternal characteristics (age, marital status, employment status, psychiatric history such as depression), paternal characteristics (age), infant characteristics (single or multiple birth, birth weight, gestational age, living with siblings), and household characteristics. | Y | 29 |
| ED visit | Depression (CES-D >= 16) | Women in pediatric ED of University of Michigan health system | Not given | n = 176 | ER visits in past 6 months: OR = 2.90 (1.18–8.70) Missed pediatric outpatient visits in past year: OR = 2.91 (1.18–8.70) | Cross-sectional study using multivariate regression models to examine the association of maternal depression in mothers of young children and child health care use, controlling for the presence of child chronic illness, child age, maternal age, years of maternal education, insurance status. | Y | 30 |
| ED visit | Maternal depression (diagnoses based on medical and claim records that are identified with the ICD-9 depression codes) | Kaiser Permanente membership system | 1997–2002 | n = 69,665 | Adjusted rate ratios for ED visits = 1.23 (children 3–11 months), 1.31 (children 1–2 years), and 1.15 (children 3–5 years) | Retrospective, matched-cohort design, examining associations between parental depression and child health care use, controlling for child’s gender, number of parents, and a risk-adjustment variable to account for possible morbidity differences between exposed and unexposed children. We focus on adjusted rate ratios for children under 5. | Y | 31 |
| Well-child care visits | Maternal depression at 2–4 months post-delivery | National Evaluation of Healthy Steps for Young Children (HS) | 1996–1998 | n = 4,896 | Age-appropriate well-child visits at 12 months: OR = 0.80 (0.67–0.95) | Logistic regression for dichotomous outcomes and Poisson regression for count outcomes) were used to estimate the effect of maternal depressive symptoms on children’s receipt of care. Models were adjusted for baseline demographic characteristics, child health status, and other potential confounders. | Y | 32 |

**Sources/notes:**

SOURCE: Scientific literature and national databases (see specific sources below).

NOTES: * indicates that the majority of papers in the systematic review demonstrated statistically significant effects.

CESD = Center for Epidemiologic Studies Depression Scale; DSM = Diagnostic and Statistical Manual; ED = emergency department; EPDS = Edinburgh Postnatal Depression Scale; ED = emergency department; ICD = International Classification of Diseases; NHANES = National Health and Nutrition Examination Survey; OR = odds ratio; PPD = post-delivery depression; PRISMA = Preferred Reporting Items for Systematic Reviews and Meta-Analyses; SDQ = Strengths and Difficulties Questionnaire; SNAP = Supplemental Nutrition Assistance Program; STAI = State Trait Anxiety Inventory; TANF = Temporary Assistance for Needy Families; WIC = Women, Infants, and Children Program

Supplementary Table 3

**Caption:** Prevalence of MMHCs

| **Outcome** | **Data source** | **Study population** | | **Data year** | **Subgroup** | | **Point estimate**  **(95% CI)** | |
| --- | --- | --- | --- | --- | --- | --- | --- | --- |
| Post-delivery depression and post-delivery depressive symptoms | Pregnancy Risk Assessment Monitoring System | | Women across 31 states with a recent live birth (surveyed 2–6 months post-delivery) | 2018 | | National | | 13.2%  (12.6%–13.8%) |
|  |  |  |  |  |  | Non-Hispanic White | | 11.4%  (10.7%–12.1%) |
|  |  |  |  |  |  | Non-Hispanic Black | | 18.2%  (16.5%–19.9%) |
|  |  |  |  |  |  | Hispanic | | 12.0%  (10.8%–13.2%) |
|  |  |  |  |  |  | Medicaid | | 17.2%  (16.3%–18.2%) |

**Sources/notes:**

SOURCE: Bauman BL, Ko JY, Cox S, D'Angelo DV, Warner L, Folger S, et al. Vital signs: post-delivery depressive symptoms and provider discussions about perinatal depression - United States, 2018. Morb Mortal Wkly Rep. 2020;69(19):575-81.

Supplementary Table 4

**Caption:** Studies and Data Sources Used to Inform the Cost Estimates Used in the Main Model

| **Parameter** | **Sample Years** | **Estimate**  **(in 2019 $)** | **Methodology** | **Citation/**  **Data Source** |
| --- | --- | --- | --- | --- |
| Maternal outcomes | | | | |
| Cost per unemployed woman | 2019 | 40,144 | To calculate the cost per unemployed woman, we assumed that women are paid for working 52 weeks per year. We multiplied 52 weeks by the median weekly earnings data from the Bureau of Labor Statistics (BLS). | 33 |
| Annual cost per case of suicide | 2013 | 43,550 | We obtained the direct costs of suicide, including ambulance transport, a coroner or medical examination, an emergency department visit, inpatient hospitalization, and nursing home care from Shepard et al (2017). We calculated the indirect costs of suicide by estimating the annual salary of women with children under age 6, based on data from BLS. | 33, 34 |
| Annual cost per case of preeclampsia | 2011-2015 | 18,016 | We calculated the cost of pre-eclampsia for mothers and their infants combined, where the infant is born full-term (37 weeks or greater), to avoid overlap in costs with preterm birth.  The individual maternal and infant costs were obtained from Hao et al (2019), which used a matched control cost-of-illness methodology to estimate the direct health care cost burden of pre-eclampsia, controlling for maternal age, parity, obesity status, and mean Charlson Comorbidity Index scores. | 35 |
| Incremental cost per case of cesarean delivery | 2010 | 12,179 | We calculated the incremental cost of delivery via cesarean section as the additional medical cost of a cesarean delivery beyond that for a vaginal delivery. | 36 |
| Daily cost per inpatient stay | 2017 | 2,416 | We used the daily cost of an inpatient stay as a proxy for the cost of an additional day of a peripartum stay. | 37 |
| Cost per SNAP recipient | 2016 | 1,720 | We calculated the cost per person on SNAP from the Texas-specific estimate of monthly administrative costs, multiplied by 12 to get an estimate of annual per capita administrative costs. We added this to the total per capita annual benefit cost to obtain an estimate of the total annual cost. | 38 |
| Cost per WIC recipient | 2019 | 605 | We calculated the cost per person on WIC from the total issuance and administrative costs of WIC, divided by the total number of participants. | 39, 40 41 |
| Cost per Medicaid beneficiary | 2014 | 7,766 | This estimate represents personal health care spending among all full- or partial-benefit Medicaid beneficiaries in 2014. The estimate reflects all health care goods and services consumed by Medicaid beneficiaries but excludes government administrative costs and the costs of government public health activities and investment. | 42, 43 |
| Cost per TANF recipient | 2016 | 10,374 | We calculated the cost per TANF case by dividing total federal TANF and state maintenance-of-effort expenditures, including both services and administrative costs, by the average number of TANF recipients in federal fiscal year 2016. | 44, 45 |
| Child outcomes | | | | |
| Incremental cost per infant with preterm birth | 2016 | 49,758 | We based the incremental cost per infant born pre-term on Waitzman & Jalali (2019). Their report considered medical care for the affected child, maternal delivery costs, early intervention services, special education services, devices, and lost labor market productivity. To avoid double-counting costs and to adhere to our model timeframe (from conception to age 5 of the cohort), we included only medical care costs and early intervention costs incurred by the child from birth to age 5. These costs are counted only once in the model. | 46 |
| Incremental cost per infant due to suboptimal breastfeeding | 2007 | 1,987 | We based the incremental cost due to suboptimal breastfeeding on Bartick & Reinhold (2010), which estimated the excess cost of suboptimal breastfeeding, relative to the costs if 80% of US families could comply with the recommendation to exclusively breastfeed for 6 months. Excess costs included necrotizing enterocolitis, otitis media, gastroenteritis, hospitalization for lower respiratory tract infections, atopic dermatitis, SIDs, childhood asthma, childhood leukemia, type 1 diabetes mellitus, and childhood obesity. To avoid double-counting, we excluded the costs due to SIDS, asthma, and childhood obesity. | 47 |
| Annual cost per case of SIDS | 2010 | 22,014 | We used Fox et al’s estimate of the economic costs associated with child death. Cost components include funeral expenditures, the value of outside help over the course of 6 months, out-of-pocket prescription costs, and the costs of absenteeism and presenteeism due to parental bereavement. | 48 |
| Incremental annual cost per child with behavioral and developmental disorders | 2005–2006 | 12,990 | We based our estimate on Beecham et al (2014), who examined the costs of child mental illness among young children. Incremental costs include health and mental health care, education, social care, parents’ out-of-pocket expenses, parents’ absence from work, and accommodation (excluding parental home). | 49 |
| Incremental annual cost per child with obesity | 2012 | 248 | We used Finkelstein et al’s (2014) estimate of the incremental lifetime medical cost of an obese child relative to a normal weight child who maintains normal weight through adulthood, divided by an average life expectancy of 78.2 years. | 50 |
| Incremental annual cost per child with asthma | 2010 | 3,056 | We based the cost of asthma on Sullivan et al 2017, which presented a cross-sectional retrospective analysis of school-aged children (ages 6–17 years) in the nationally representative 2007–2013 Medical Expenditure Panel Survey. All-cause health care expenditures of school-aged children with asthma were compared with school-aged children without asthma, controlling for sociodemographic characteristics and comorbidities. Expenditures included medical, ED, inpatient, outpatient, and pharmacy costs. We excluded the cost of ED visits to avoid double-counting. | 51 |
| Annual cost per child injury | 2010 | 8,018 | We averaged the lifetime cost of a nonfatal injury that resulted in a hospitalization to children ages 0 through 5 who were hospitalized by an average life expectancy of 78.2 years to estimate the annual cost per child injury. Hospitalization costs are considered separately in the CDC database we used (Web-based Injury Statistics Query and Reporting System [WISQARS]). | 52 |
| Cost per ED visit for child | 2014 | 714 | We used an estimate of the average expenses per child under age 5 living in Census Region 3 (South). Expenses include both the ER facility fee and separately-billed doctor expenditures. To obtain a reasonable estimate of mean ED expenditures and remove noise created by surveyed patients who did not visit the ED, we restricted the data to expenditures of at least $100. | 53 |
| Cost per well-child care visit | 2015 | 514 | We used an estimate of the median expenses for an office-based provider visit for a child ages 0 through 5 with a perceived health status of Excellent, Very Good, Good, or Fair. | 54 |

**Sources/notes:**

SOURCE: Scientific literature and national databases (see specific sources below).

NOTES: ED = emergency department; SNAP = Supplemental Nutrition Assistance Program; TANF = Temporary Assistance for Needy Families; WIC = Women, Infants, and Children Program

Supplementary Table 5

**Caption:** Model Inputs (main model): Parameters and Costs Used to Estimate the Economic Impact of Untreated MMHCs Among 2019 Births

| **Parameter** | **Point estimate**  **(Range for sensitivity analyses)** | **Source** |
| --- | --- | --- |
| Baseline demographic characteristics | | |
| Number of births^a,c^ | 377,397 | 55 |
| Number of pregnancies^a,b,c^ | 595,881 | 56, 57 |
| Prevalence of MMHCs (%)^b^ | 13.2 (12.6-13.8) | 58 |
| Other inputs |  |  |
| Medical care inflation (%)^b^ | 4.53 | 59 |
| Discount rate (%)^b^ | 3.00 | 60 |
| Women who do not achieve remission without treatment by the end of the first year post-delivery (%)^b,c^ | 33.3 (20.0-60.0) | 1 |
| Maternal outcomes | | |
| Maternal productivity |  |  |
| Labor force participation among women with children aged younger than 6 years (%)^b,c^ | 62.0 | 61 |
| Per capita expected cost of job absenteeism ($)^b,c^ | 1,104 (415-1,792) | 2, 3, 4, 5 |
| Per capita expected cost of job presenteeism ($)^b,c^ | 3,107 (410-5,804) | 2, 4, 5 |
| Baseline rate of unemployment (%)^b,c^ | 4.6 | 61 |
| Likelihood of unemployment among women with MMHCs (%) | 6.0 (5.5-6.4) | 6 |
| Cost per unemployed woman ($)^a,c^ | 40,144 | 33 |
| Suicide |  |  |
| Baseline incidence among women (%)^a,c^ | 0.0058 | 62 |
| Likelihood of suicide among women with depression (%)^b,c^ | 0.136 (0.116-0.157) | 4 |
| Annual cost per case of suicide ($)^a,c^ | 43,550 | 33, 34 |
| Maternal obstetric health |  |  |
| Baseline incidence of pre-eclampsia (%)^b,c^ | 4.7 | 63 |
| Likelihood of pre-eclampsia among women with MMHCs (%)^b,c^ | 8.8 (6.9-10.6) | 8, 9, 10, 11, 12 |
| Annual cost per case of pre-eclampsia ($)^b,c^ | 18,016 | 35 |
| Baseline incidence of cesarean delivery (%)^a,c^ | 34.8 | 55 |
| Likelihood of cesarean delivery among women with MMHCs (%)^b,c^ | 43.9 (41.7-45.9) | 11, 13, 14 |
| Incremental cost per case of cesarean delivery ($)^b,c^ | 12,179 | 36 |
| Average peripartum stay (days)^b,c^ | 2.6 | 64 |
| Average peripartum stay for women with MMHCs (days) ^b,c^ | 2.86 (2.64-3.08) | 15 |
| Daily cost per inpatient stay ($)^b,c^ | 2,416 | 37 |
| Maternal health expenditures |  |  |
| Individual out-of-pocket expenditures for women without MMHCs ($)^b,c^ | 657 (418-697) | 3 |
| Individual out-of-pocket expenditures for women with MMHCs ($)^b,c^ | 996 (635-1,058) | 3 |
| Individual insurer expenditures for women without MMHCs ($)^b,c^ | 3,853 (2,456-4,093) | 3 |
| Individual insurer expenditures for women with MMHCs ($)^b,c^ | 5,579 (3,556-5,926) | 3 |
| Benefit receipt |  |  |
| SNAP receipt among families with children aged younger than 18 years (%)^a,c^ | 59.9 | 65 |
| Likelihood of SNAP receipt among women with MMHCs (%)^a,c^ | 60.1 (60.0-60.2) | 16 |
| Cost per person receiving SNAP benefits ($)^a,c^ | 1,720 | 38 |
| WIC receipt among women with children under age 5 (%)^a,c^ | 29.4 | 66 |
| Likelihood of WIC receipt among women with MMHCs (%)^a,c^ | 29.6 (29.5-29.7) | 16 |
| Cost per person receiving WIC benefits ($)^a,c^ | 605 | 39, 40, 41 |
| Medicaid receipt among women aged 15–44 years (%)^a,c^ | 47.5 | 67 |
| Likelihood of Medicaid receipt among women with MMHCs (%)^a,c^ | 47.75 (47.65-47.84) | 16 |
| Cost per person receiving Medicaid benefits ($)^a,c^ | 7,766 | 42, 43 |
| TANF receipt among families with children aged younger than 18 years (%)^a,c^ | 0.7 | 68, 69 |
| Likelihood of TANF receipt among women with MMHCs (%)^a,c^ | 0.9 (0.8-1.0) | 16 |
| Cost per person receiving TANF benefits ($)^a,c^ | 10,374 | 44, 45 |
| Child outcomes | | |
| Preterm birth |  |  |
| Baseline incidence (%)^a,c^ | 11.0 | 55 |
| Likelihood among infants born to women with MMHCs (%)^a,c^ | 26.0 (8.1-38.1) | 9, 17, 18 |
| Incremental cost per infant with preterm birth ($)^b,c^ | 49,758 | 46 |
| Suboptimal breastfeeding |  |  |
| Baseline prevalence of exclusive breastfeeding through 3 months post-delivery (%)^a,c^ | 45.8 | 70 |
| Likelihood among women with MMHCs (%)^a,c^ | 40.0 (37.2-42.6) | 19 |
| Incremental cost per infant ($)^b,c^ | 1,987 | 47 |
| SIDS |  |  |
| Baseline incidence (%)^a,c^ | 0.038 | 71 |
| Likelihood among babies born to mothers with MMHCs (%)^a,c^ | 0.16 (0.1-0.2) | 20, 21 |
| Annual cost per case ($)^b,c^ | 22,014 | 48 |
| Child behavioral and developmental disorders |  |  |
| Baseline prevalence among children (%)^a,c^ | 17.3 | 72 |
| Likelihood among children born to women with MMHCs (%)^a,c^ | 31.1 (28.0-34.3) | 22 |
| Incremental annual cost per child ($)^b,c^ | 12,990 | 49 |
| Childhood obesity |  |  |
| Baseline prevalence among children aged 2–4 years (%)^a,c^ | 14.6 | 73 |
| Likelihood among children born to women with MMHCs (%)^a,c^ | 19.9 (4.6-30.9) | 23, 24, 25 |
| Incremental annual cost per child ($)^b,c^ | 248 | 50 |
| Child asthma |  |  |
| Baseline prevalence among children aged 0–4 years (%)^b,c^ | 3.8 | 74 |
| Likelihood among children born to women with MMHCs (%)^b,c^ | 7.3 (6.1-8.5) | 26, 27 |
| Incremental annual cost per child ($)^b,c^ | 3,056 | 51 |
| Child nonfatal injury |  |  |
| Baseline incidence of nonfatal injury among children aged 0–4 years (%)^b,c^ | 8.2 | 75 |
| Likelihood of injury among children born to women with MMHCs (%)^b,c^ | 10.6 (8.7-12.4) | 28, 29 |
| Annual cost per childhood injury ($)^b,c^ | 8,018 | 52 |
| Child emergency department visits |  |  |
| Baseline incidence among children aged 1–4 years (%)^b,c^ | 62.8 | 76 |
| Likelihood among children born to women with MMHCs (%)^b,c^ | 78.9 (67.5-84.4) | 30, 31 |
| Cost per visit for child ($)^a,c^ | 714 | 53 |
| Nonattendance of well-child care visits |  |  |
| Baseline likelihood among children aged 0–6 years (%)^b,c^ | 35.0 | 77 |
| Likelihood among children born to women with MMHCs (%)^b,c^ | 51.2 (35.0-61.0) | 32 |
| Cost per visit ($)^a,c^ | 514 | 54 |

**Sources/notes:**

SOURCE: Scientific literature and national databases (see specific sources below).

NOTES: MMHCs = maternal mental health conditions; SIDS = sudden infant death syndrome; SNAP = Supplemental Nutrition Assistance Program; TANF = Temporary Assistance for Needy Families; WIC = Special Supplemental Nutrition Program for Women, Infants, and Children.

^a^ Texas-specific estimate

^b^ National estimate

^c^ Estimate for all races, ethnicities, origins, and payers

Supplementary Table 6

**Caption:** Model Inputs (women enrolled in Texas Medicaid for Pregnant Women): Parameters and Costs Used to Estimate the Economic Impact of Untreated MMHCs Among 2019 Births

| **Parameter** | **Point estimate** | **Source** |
| --- | --- | --- |
| Baseline demographic characteristics | | |
| Number of births^a^ | 179,264 | 67 |
| Number of pregnancies^a^ | 283,043 | 67, 56, 57 |
| Prevalence of MMHCs (%)^a^ | 17.2 | 58 |
| Other inputs |  |  |
| Medical care inflation (%) | 4.53 | 59 |
| Discount rate (%) | 3.00 | 60 |
| Women who do not achieve remission without treatment by the end of the first year post-delivery (%) | 33.3 | 1 |
| Maternal outcomes | | |
| Maternal obstetric health |  |  |
| Baseline incidence of pre-eclampsia (%)^b,c^ | 4.9 | 63 |
| Likelihood of pre-eclampsia among women with MMHCs (%) | 9.12 | 8, 9, 10, 11, 12 |
| Annual cost per case of pre-eclampsia ($)^b^ | 18,016 | 35 |
| Baseline incidence of cesarean delivery (%)^a,c^ | 34.2 | 67 |
| Likelihood of cesarean delivery among women with MMHCs (%) | 43.2 | 11, 13, 14 |
| Incremental cost per case of cesarean delivery ($) | 5,339 | 36 |
| Average peripartum stay (days) ^b,c^ | 2.6 | 64 |
| Average peripartum stay for women with MMHCs (days) | 2.86 | 15 |
| Daily cost per inpatient stay ($) | 2,119 | 37 |
| Maternal health expenditures |  |  |
| Individual out-of-pocket expenditures for women without MMHCs ($) | 657 | 3 |
| Individual out-of-pocket expenditures for women with MMHCs ($) | 996 | 3 |
| Individual insurer expenditures for women without MMHCs ($) | 3,853 | 3 |
| Individual insurer expenditures for women with MMHCs ($) | 5,579 | 3 |
| Benefit receipt |  |  |
| Medicaid receipt among women aged 15–44 years (%) | 47.5 | 67 |
| Likelihood of Medicaid among women with MMHCs (%) | 47.74 | 16 |
| Cost per case of Medicaid ($) | 7,766 | 42, 43 |
| Child outcomes | | |
| Preterm birth |  |  |
| Baseline incidence (%)^a^ | 11.1 | 67 |
| Likelihood among infants born to women with MMHCs (%)^a^ | 26.18 | 9, 17, 18 |
| Incremental cost per infant with preterm birth ($)^a,c^ | 49,758 | 46 |
| Suboptimal breastfeeding |  |  |
| Baseline prevalence (%)^a,c^ | 45.8 | 70 |
| Likelihood among women with MMHCs (%)^b,c^ | 40.0 | 19 |
| Incremental cost per infant ($)^b,c^ | 1,987 | 47 |
| Child behavioral and developmental disorders |  |  |
| Baseline prevalence among children aged 2–8 years (%)^a,c^ | 17.3 | 72 |
| Likelihood among children born to women with MMHCs (%) | 31.1 | 22 |
| Incremental annual cost per child ($) | 12,990 | 49 |
| Childhood obesity |  |  |
| Baseline prevalence among children aged 2–5 years (%)^b,c^ | 14.6 | 73 |
| Likelihood among children born to women with MMHCs (%) | 19.9 | 23, 24, 25 |
| Incremental annual cost per child ($) | 248 | 50 |
| Child asthma |  |  |
| Baseline prevalence among children aged 0–4 years (%)^b,c^ | 3.8 | 74 |
| Likelihood among children born to women with MMHCs (%) | 7.32 | 26, 27 |
| Incremental annual cost per child ($) | 3,056 | 49 |
| Child nonfatal injury |  |  |
| Baseline incidence of nonfatal injury among children aged 0–4 years (%)^b,c^ | 8.2 | 75 |
| Likelihood of injury among children born to women with MMHCs (%) | 10.58 | 28, 29 |
| Annual cost per childhood injury ($) | 8,018 | 52 |
| Child emergency department visits |  |  |
| Baseline incidence among children aged 1–4 years (%)^b,c^ | 62.8 | 76 |
| Likelihood among children born to women with MMHCs (%) | 78.90 | 30, 31 |
| Cost per child emergency department visit ($)^a^ | 383 | 53 |
| Nonattendance of well-child care visits |  |  |
| Baseline likelihood among children aged 0–6 years (%)^b,c^ | 35.0 | 77 |
| Likelihood among children born to women with MMHCs (%) | 51.2 | 32 |
| Cost per visit ($)^a^ | 156 | 54 |

**Sources/notes:**

SOURCE: Scientific literature and national databases (see specific sources below).

NOTES: MMHCs = maternal mental health conditions; SIDS = sudden infant death syndrome; SNAP = Supplemental Nutrition Assistance Program; TANF = Temporary Assistance for Needy Families; WIC = Special Supplemental Nutrition Program for Women, Infants, and Children.

^a^ Texas-specific estimate

^b^ National estimate

^c^ Estimate for all races, ethnicities, origins, and payers

Supplementary Table 7

Caption: Model results for costs of untreated MMHCs (in millions of dollars) for the 2019 birth cohort: Non-Hispanic White mothers

| **Outcomes** | **Total** | **Year 0** | **Year 1** | **Year 2** | **Year 3** | **Year 4** | **Year 5** |
| --- | --- | --- | --- | --- | --- | --- | --- |
| Maternal costs | | | | | | | |
| Productivity losses | 169.5 | 61.9 | 21.5 | 22.5 | 23.5 | 24.6 | 25.7 |
| Suicide | 8.0 | 2.9 | 1.0 | 1.1 | 1.1 | 1.2 | 1.2 |
| Preeclampsia^a^ | 8.8 | 8.8 | 0.0 | 0.0 | 0.0 | 0.0 | 0.0 |
| Cesarean delivery^a^ | 14.7 | 14.7 | 0.0 | 0.0 | 0.0 | 0.0 | 0.0 |
| Peripartum stay^a^ | 8.7 | 8.7 | 0.0 | 0.0 | 0.0 | 0.0 | 0.0 |
| Non-obstetric health expenditures | 122.7 | 44.8 | 15.6 | 16.3 | 17.0 | 17.8 | 18.6 |
| Benefit receipt | 5.6 | 1.0 | 1.0 | 1.0 | 1.0 | 1.0 | 1.0 |
| Child costs | | | | | | | |
| Preterm birth^a^ | 89.2 | 89.2 | 0.0 | 0.0 | 0.0 | 0.0 | 0.0 |
| Suboptimal breastfeeding^a^ | 1.6 | 1.6 | 0.0 | 0.0 | 0.0 | 0.0 | 0.0 |
| SIDS | 0.4 | 0.4 | 0.0 | 0.0 | 0.0 | 0.0 | 0.0 |
| Behavioral and developmental disorders | 154.9 | 24.9 | 26.0 | 27.2 | 28.4 | 29.7 | 31.0 |
| Obesity | 1.1 | 0.2 | 0.2 | 0.2 | 0.2 | 0.2 | 0.2 |
| Asthma | 4.5 | 0.8 | 0.8 | 0.8 | 0.8 | 0.8 | 0.8 |
| Injury | 7.1 | 2.6 | 0.9 | 0.9 | 1.0 | 1.0 | 1.1 |
| Emergency department visits | 5.3 | 1.9 | 0.7 | 0.7 | 0.7 | 0.8 | 0.8 |
| Non-attendance of well-child care visits | -3.3 | -1.2 | -0.4 | -0.4 | -0.5 | -0.5 | -0.5 |
| Total societal costs for one birth cohort (millions of $) | **599** | **263** | **67** | **70** | **73** | **77** | **80** |
| Cost per mother–child pair with an MMHC in the first year post-delivery ($) | **18,941** |  |  |  |  |  |  |
| Cost per mother–child pair with an MMHC in the first two years post-delivery ($) | **23,784** |  |  |  |  |  |  |
| Cost per mother–child pair with an MMHC, averaged over the first two years post-delivery ($) | **11,892** |  |  |  |  |  |  |
| Cost per mother–child pair with an MMHC in the first five years post-delivery ($) | **43,106** |  |  |  |  |  |  |
| Cost per mother–child pair with an MMHC, averaged over the six years from conception through the first five years post-delivery ($) | **7,184** |  |  |  |  |  |  |
| **Sources/notes:**  SOURCE Authors’ analysis.  NOTES ^a^ Costs only apply to Year 0, the year of conception and birth. We assumed that other costs are incurred annually through Year 5.  MMHCs = maternal mental health conditions; SIDS = sudden infant death syndrome. | | | | | | | |

Supplementary Table 8

**Caption:** Model results for costs of untreated MMHCs (in millions of dollars) for the 2019 birth cohort: Non-Hispanic Black mothers

| **Outcome** | **Total** | **Year 0** | **Year 1** | **Year 2** | **Year 3** | **Year 4** | **Year 5** |
| --- | --- | --- | --- | --- | --- | --- | --- |
| **Maternal costs** | | | | | | | |
| Productivity losses | 189.4 | 69.1 | 24.1 | 25.1 | 26.3 | 27.5 | 28.7 |
| Suicide | 2.4 | 0.9 | 0.3 | 0.3 | 0.3 | 0.4 | 0.4 |
| Preeclampsia^a^ | 8.8 | 8.8 | 0.0 | 0.0 | 0.0 | 0.0 | 0.0 |
| Cesarean delivery^a^ | 9.5 | 9.5 | 0.0 | 0.0 | 0.0 | 0.0 | 0.0 |
| Peripartum stay^a^ | 5.3 | 5.3 | 0.0 | 0.0 | 0.0 | 0.0 | 0.0 |
| Non-obstetric health expenditures | 117.5 | 42.9 | 14.9 | 15.6 | 16.3 | 17.1 | 17.8 |
| Benefit receipt | 5.0 | 0.9 | 0.9 | 0.9 | 0.9 | 0.9 | 0.9 |
| **Child costs** | | | | | | | |
| Preterm birth^a^ | 74.0 | 74.0 | 0.0 | 0.0 | 0.0 | 0.0 | 0.0 |
| Suboptimal breastfeeding^a^ | 1.0 | 1.0 | 0.0 | 0.0 | 0.0 | 0.0 | 0.0 |
| SIDS | 0.5 | 0.5 | 0.0 | 0.0 | 0.0 | 0.0 | 0.0 |
| Behavioral and developmental disorders | 93.9 | 15.1 | 15.8 | 16.5 | 17.2 | 18.0 | 18.8 |
| Obesity | 0.6 | 0.1 | 0.1 | 0.1 | 0.1 | 0.1 | 0.1 |
| Asthma | 11.4 | 1.8 | 1.9 | 2.0 | 2.1 | 2.2 | 2.3 |
| Injury | 3.3 | 1.2 | 0.4 | 0.4 | 0.5 | 0.5 | 0.5 |
| Emergency department visits | 0.8 | 0.3 | 0.1 | 0.1 | 0.1 | 0.1 | 0.1 |
| Non-attendance of well-child care visits | -2.0 | -0.7 | -0.2 | -0.3 | -0.3 | -0.3 | -0.3 |
| Total societal costs for one birth cohort (millions of $) | **521** | **231** | **58** | **61** | **64** | **67** | **69** |
| Cost per mother–child pair with an MMHC in the first year post-delivery ($) | **27,308** |  |  |  |  |  |  |
| Cost per mother–child pair with an MMHC in the first two years post-delivery ($) | **34,209** |  |  |  |  |  |  |
| Cost per mother–child pair with an MMHC, averaged over the first two years post-delivery ($) | **17,105** |  |  |  |  |  |  |
| Cost per mother–child pair with an MMHC in the first five years post-delivery ($) | **61,671** |  |  |  |  |  |  |
| Cost per mother–child pair with an MMHC, averaged over the six years from conception through the first five years post-delivery ($) | **10,279** |  |  |  |  |  |  |
| **Sources/notes:**  SOURCE: Authors’ analysis.  NOTES: ^a^ Costs only apply to Year 0, the year of conception and birth. We assumed that other costs are incurred annually through Year 5.  MMHCs = maternal mental health conditions; SIDS = sudden infant death syndrome. | | | | | | | |

Supplementary Table 9

**Caption:** Model results for costs of untreated MMHCs (in millions of dollars) for the 2019 birth cohort: Hispanic mothers

| **Outcomes** | **Total** | **Year 0** | **Year 1** | **Year 2** | **Year 3** | **Year 4** | **Year 5** |  |
| --- | --- | --- | --- | --- | --- | --- | --- | --- |
| Maternal costs | | | | | | | | |
| Productivity losses | 229.0 | 83.6 | 29.1 | 30.4 | 31.8 | 33.2 | 34.7 |  |
| Suicide | 3.6 | 1.3 | 0.5 | 0.5 | 0.5 | 0.5 | 0.5 |  |
| Preeclampsia^a^ | 16.6 | 16.6 | 0.0 | 0.0 | 0.0 | 0.0 | 0.0 |  |
| Cesarean delivery^a^ | 22.7 | 22.7 | 0.0 | 0.0 | 0.0 | 0.0 | 0.0 |  |
| Peripartum stay^a^ | 13.5 | 13.5 | 0.0 | 0.0 | 0.0 | 0.0 | 0.0 |  |
| Non-obstetric health expenditures | 211.0 | 77.0 | 26.8 | 28.0 | 29.3 | 30.6 | 32.0 |  |
| Benefit receipt | 10.3 | 1.6 | 1.7 | 1.8 | 1.9 | 2.0 | 2.1 |  |
| Child costs | | | | | | | | |
| Preterm birth^a^ | 149.2 | 149.2 | 0.0 | 0.0 | 0.0 | 0.0 | 0.0 |  |
| Suboptimal breastfeeding^a^ | 2.5 | 2.5 | 0.0 | 0.0 | 0.0 | 0.0 | 0.0 |  |
| SIDS | 0.5 | 0.5 | 0.0 | 0.0 | 0.0 | 0.0 | 0.0 |  |
| Behavioral and developmental disorders | 238.9 | 38.4 | 40.1 | 41.9 | 43.8 | 45.8 | 47.9 |  |
| Obesity | 2.2 | 0.4 | 0.4 | 0.4 | 0.4 | 0.4 | 0.4 |  |
| Asthma | 17.5 | 2.9 | 3.0 | 3.1 | 3.2 | 3.3 | 3.4 |  |
| Injury | 7.1 | 2.6 | 0.9 | 0.9 | 1.0 | 1.0 | 1.1 |  |
| Emergency department visits | 8.1 | 3.0 | 1.0 | 1.1 | 1.1 | 1.2 | 1.2 |  |
| Non-attendance of well-child care visits | -4.9 | -1.8 | -0.6 | -0.7 | -0.7 | -0.7 | -0.7 |  |
| Total societal costs for one birth cohort (millions of $) | **928** | **414** | **103** | **107** | **112** | **117** | **123** |  |
| Cost per mother–child pair with an MMHC in the first year post-delivery ($) | **19,327** |  |  |  |  |  |  |  |
| Cost per mother–child pair with an MMHC in the first two years post-delivery ($) | **24,131** |  |  |  |  |  |  |  |
| Cost per mother–child pair with an MMHC, averaged over the first two years post-delivery ($) | **12,066** |  |  |  |  |  |  |  |
| Cost per mother–child pair with an MMHC in the first five years post-delivery ($) | **43,322** |  |  |  |  |  |  |  |
| Cost per mother–child pair with an MMHC, averaged over the six years from conception through the first five years post-delivery ($) | **7,220** |  |  |  |  |  |  |  |
| **Sources/notes:**  SOURCE Authors’ analysis.  NOTES ^a^ Costs only apply to Year 0, the year of conception and birth. We assumed that other costs are incurred annually through Year 5. MMHCs = maternal mental health conditions; SIDS = sudden infant death syndrome. | | | | | | | |  |

Supplementary Table 10

**Caption:** Model Inputs (Subgroup analysis by maternal race and ethnicity): Parameters and Costs Used to Estimate the Economic Impact of Untreated MMHCs Among 2019 Births

|  | **Point Estimates** | | |  |
| --- | --- | --- | --- | --- |
| **Parameters** | **Non-Hispanic White Mothers** | **Non-Hispanic Black Mothers** | **Hispanic Mothers** | **Source** |
| Baseline demographic characteristics | | | | |
| Number of births^a^ | 121,899 | 46,420 | 178,509 | 55, 78 |
| Number of pregnancies^a^ | 190,384 | 114,062 | 310,530 | 56, 79 |
| Prevalence of MMHCs (%)^a^ | 11.4 | 18.2 | 12.0 | 58 |
| Other inputs |  |  |  |  |
| Medical care inflation (%)^b,c^ | 4.53 | 4.53 | 4.53 | 59 |
| Discount rate (%)^b,c^ | 3.00 | 3.00 | 3.00 | 60 |
| Women who do not achieve remission without treatment by the end of the first year post-delivery (%)^b,c^ | 33.3 | 33.3 | 33.3 | 1 |
| Maternal outcomes | | | | |
| Maternal productivity |  |  |  |  |
| Labor force participation among women with children aged younger than 6 years (%) | 62.0 | 71.0 | 49.0 | 61 |
| Per capita expected cost of job absenteeism ($)^b,c^ | 1,104 | 1,104 | 1,104 | 2, 3, 4, 5 |
| Per capita expected cost of job presenteeism ($)^b,c^ | 3,107 | 3,107 | 3,107 | 2, 4, 5 |
| Baseline rate of unemployment (%)^b^ | 4.0 | 10.0 | 7.0 | 61 |
| Likelihood of unemployment among women with MMHCs (%)^a^ | 5.2 | 13.0 | 9.1 | 6 |
| Cost per unemployed woman ($)^a^ | 50,555 | 38,891 | 30,685 | 33 |
| Suicide |  |  |  |  |
| Baseline incidence among women (%)^a^ | 0.0109 | 0.0046 | 0.0045 | 62 |
| Likelihood of suicide among women with depression (%)^a^ | 0.256 | 0.108 | 0.106 | 4 |
| Annual cost per case of suicide ($)^a^ | 53,961 | 42,297 | 34,091 | 33, 34 |
| Maternal obstetric health |  |  |  |  |
| Baseline incidence of pre-eclampsia (%)^b^ | 4.0 | 7.0 | 5.0 | 63 |
| Likelihood of pre-eclampsia among women with MMHCs (%) | 7.5 | 12.8 | 9.3 | 8, 9, 10, 11, 12 |
| Annual cost per case of pre-eclampsia ($)^b,c^ | 18,016 | 18,016 | 18,016 | 35 |
| Baseline incidence of cesarean delivery (%)^b^ | 30.7 | 36.0 | 31.3 | 55 |
| Likelihood of cesarean delivery among women with MMHCs (%) | 39.7 | 45.2 | 39.7 | 11, 13, 14 |
| Incremental cost per case of cesarean delivery ($)^b,c^ | 12,179 | 12,179 | 12,179 | 36 |
| Average peripartum stay (days)^b,c^ | 2.6 | 2.6 | 2.6 | 64 |
| Average peripartum stay for women with MMHCs (days) ^b,c^ | 2.86 | 2.86 | 2.86 | 15 |
| Daily cost per inpatient stay ($)^b,c^ | 2,416 | 2,416 | 2,416 | 37 |
| Maternal health expenditures |  |  |  |  |
| Individual out-of-pocket expenditures for women without MMHCs ($)^b,c^ | 657 | 657 | 657 | 3 |
| Individual out-of-pocket expenditures for women with MMHCs ($)^b,c^ | 996 | 996 | 996 | 3 |
| Individual insurer expenditures for women without MMHCs ($)^b,c^ | 3,853 | 3,853 | 3,853 | 3 |
| Individual insurer expenditures for women with MMHCs ($)^b,c^ | 5,579 | 5,579 | 5,579 | 3 |
| Benefit receipt |  |  |  |  |
| SNAP receipt among families with children aged younger than 18 years (%)^a^ | 24.0 | 23.0 | 49.0 | 65 |
| Likelihood of SNAP receipt among women with MMHCs (%)^a^ | 24.0 | 23.0 | 49.0 | 16 |
| Cost per person receiving SNAP benefits ($)^a,c^ | 1,720 | 1,720 | 1,720 | 38 |
| WIC receipt among women with children under age 5 (%)^b^ | 17.0 | 39.0 | 41.0 | 66 |
| Likelihood of WIC receipt among women with MMHCs (%)^b^ | 17.2 | 39.2 | 41.2 | 16 |
| Cost per person receiving WIC benefits ($)^a,c^ | 605 | 605 | 605 | 39, 40, 41 |
| Medicaid receipt among women aged 15–44 years (%)^b^ | 29.0 | 65.0 | 59.0 | 80 |
| Likelihood of Medicaid receipt among women with MMHCs (%)^b^ | 29.2 | 65.2 | 59.2 | 16 |
| Cost per person receiving Medicaid benefits ($)^b,c^ | 7,766 | 7,766 | 7,766 | 42, 43 |
| TANF receipt among families with children aged younger than 18 years (%)^b^ | 0.7 | 0.2 | 0.4 | 53, 54 |
| Likelihood of TANF receipt among women with MMHCs (%)^b^ | 0.9 | 0.4 | 0.6 | 16 |
| Cost per person receiving TANF benefits ($)^a,c^ | 10,374 | 10,374 | 10,374 | 44, 45 |
| Child outcomes | | | | |
| Preterm birth |  |  |  |  |
| Baseline incidence (%)^a^ | 9.0 | 14.0 | 10.0 | 55 |
| Probability among infants born to women with MMHCs (%) | 21.9 | 31.6 | 24.0 | 9, 17, 18 |
| Incremental cost per infant with preterm birth ($)^b,c^ | 49,758 | 49,758 | 49,758 | 46 |
| Suboptimal breastfeeding |  |  |  |  |
| Baseline prevalence of exclusive breastfeeding through 3 months post-delivery (%)^a^ | 54.0 | 43.0 | 50.0 | 81 |
| Likelihood among women with MMHCs (%) | 48.1 | 37.3 | 44.1 | 19 |
| Incremental cost per infant ($)^b,c^ | 1,987 | 1,987 | 1,987 | 47 |
| SIDS |  |  |  |  |
| Baseline incidence (%)^a^ | 0.042 | 0.077 | 0.026 | 71 |
| Probability among babies born to mothers with MMHCs (%) | 0.17 | 0.31 | 0.11 | 20, 21 |
| Annual cost per case ($)^b,c^ | 22,014 | 22,014 | 22,014 | 48 |
| Child behavioral and developmental disorders |  |  |  |  |
| Baseline prevalence among children aged 2–8 years (%)^a,c^ | 17.3 | 17.3 | 17.3 | 72 |
| Likelihood among children born to women with MMHCs (%) | 31.1 | 31.1 | 31.1 | 22 |
| Incremental annual cost per child ($)^b,c^ | 12,990 | 12,990 | 12,990 | 49 |
| Childhood obesity |  |  |  |  |
| Baseline prevalence among children aged 2–5 years (%)^a^ | 17.0 | 18.0 | 25.0 | 82 |
| Likelihood among children born to women with MMHCs (%) | 22.9 | 24.1 | 32.6 | 23, 24, 25 |
| Incremental annual cost per child ($)^b,c^ | 248 | 248 | 248 | 50 |
| Child asthma |  |  |  |  |
| Baseline prevalence among children aged 0–4 years (%)^b^ | 2.0 | 8.0 | 5.0 | 74 |
| Likelihood among children born to women with MMHCs (%) | 3.9 | 14.8 | 9.5 | 26, 27 |
| Incremental annual cost per child ($)^b,c^ | 3,056 | 3,056 | 3,056 | 51 |
| Child nonfatal injury |  |  |  |  |
| Baseline incidence of nonfatal injury among children aged 0–4 years (%)^b^ | 8.0 | 6.0 | 5.0 | 52 |
| Likelihood of injury among children born to women with MMHCs (%) | 10.3 | 7.8 | 6.5 | 26, 27 |
| Annual cost per childhood injury ($)^b,c^ | 8,018 | 8,018 | 8,018 | 51 |
| Child emergency department visits |  |  |  |  |
| Baseline incidence among children aged 1–4 years (%) | 42.0 | 89.0 | 38.0 | 52 |
| Likelihood among children born to women with MMHCs (%) | 61.6 | 94.7 | 57.6 | 30, 31 |
| Cost per visit for child ($)^a,c^ | 714 | 714 | 714 | 53 |
| Nonattendance of well-child care visits |  |  |  |  |
| Baseline likelihood among children aged 0–6 years (%)^b,c^ | 35.0 | 35.0 | 35.0 | 77 |
| Likelihood among children born to women with MMHCs (%)^b,c^ | 51.2 | 51.2 | 51.2 | 32 |
| Cost per visit ($)^a,c^ | 514 | 514 | 514 | 54 |
| **Sources/notes:**  SOURCE: Scientific literature and national databases (see specific sources below).  NOTES: MMHCs = maternal mental health conditions; SIDS = sudden infant death syndrome; SNAP = Supplemental Nutrition Assistance Program; TANF = Temporary Assistance for Needy Families; WIC = Special Supplemental Nutrition Program for Women, Infants, and Children.  ^a^ Texas-specific estimate  ^b^ National estimate  ^c^ Estimate for all races, ethnicities, origins, and payers | | | | |

References for supplementary files

1. Vliegen N, Casalin S, Luyten P. The course of post-delivery depression: a review of longitudinal studies. Harv Rev Psychiatry. 2014;22(1):1-22.

2. Evans-Lacko S, Knapp M. Global patterns of workplace productivity for people with depression: absenteeism and presenteeism costs across eight diverse countries. Soc Psychiatry Psychiatr Epidemiol. 2016;55(11):1525-37.

3. Ammerman RT, Chen J, Mallow PJ, Rizzo JA, Folger AT, Van Ginkel JB. Annual direct health care expenditures and employee absenteeism costs in high-risk, low income mothers with major depression. J Affect Disord. 2016;190:386-94.

4. Rost K, Smith JL, Dickinson M. The effect of improving primary care depression management on employee absenteeism and productivity. A randomized trial. Med Care. 2004;42(12):1202-10.

5. Greenberg PE, Fournier AA, Sisitsky T, Pike CT, Kessler RC. The economic impact of adults with major depressive disorder in the united states (2005 and 2010). J Clin Psychiatry. 2015;76(2):155-62.

6. Lerner D, Henke RM. what does research tell us about depression, job performance, and work productivity? J Occup Environ Med. 2008;50(4):401–10.

7. Ösby U, Brandt L, Correia N, Ekbom A, Sparen P. Excess mortality in bipolar and unipolar disorder in Sweden. Arch Gen Psychiatry. 2001;58(9):844-50.

8. Grigoriadis S, Graves L, Peer M, Mamisashvili L, Tomlinson G, Vigod SN, et al. Maternal anxiety during pregnancy and the association with adverse perinatal outcomes: systematic review and meta-analysis. J Clin Psychiatry. 2018;79(5): 17r12011.

9. Heun-Johnson H, Seabury SA, Menchine M, Claudius I, Axeen S, Lakshmanan A. Association between maternal serious mental illness and adverse birth outcomes. J Perinatol. 2019;39(5):737-45.

10. McKee K, Admon LK, Winkelman T, Muzik M, Hall S, Dalton VK, et al. Perinatal mood and anxiety disorders, serious mental illness, and delivery-related health outcomes, United States, 2006–2015. BMC Womens Health. 2020;20(150).

11. Bansil P, Kuklina EV, Meikle SF, Posner SF, Kourtis AP, Ellington SR, et al. Maternal and fetal outcomes among women with depression. J Womens Health. 2010;19(2): 329-34.

12. Qiu C, Williams MA, Calderon-Margalit R, Cripe SM, Sorensen TK. Pre-eclampsia risk in relation to maternal mood and anxiety disorders diagnosed before or during early pregnancy. Am J Hypertens. 2009;22(4):397-402.

13. Ogunyemi D, Jovanovski A, Liu J, Friedman P, Sugiyama N, Creps J, Madan I. The contribution of untreated and treated anxiety and depression to prenatal, intrapartum, and neonatal outcomes. AJP reports. 2018;8(3):e146-e157.

14. Paul IM, Downs DS, Schaefer EW, Beiler JS, Weisman CS. Post-delivery anxiety and maternal-infant health outcomes. Pediatrics. 2013;131(4): e1218-e1224.

15. Lancaster CA, Flynn HA, Johnson TRB, Marcus SM, Davis MM. Peripartum length of stay for women with depressive symptoms during pregnancy. J Womens Health. 2010;19(1):31-7.

16. Noonan K, Corman H, Reichman NE. Effects of maternal depression on family food insecurity. Econ Hum Biol, 2016;22:201-15.

17. Adhikari K, Patten SB, Lee S, Metcalfe A. Risk of adverse perinatal outcomes among women with pharmacologically treated and untreated depression during pregnancy: a retrospective cohort study. Paediatr Perinat Epidemiol, 2019;33:323-31.

18. Jarde A, Morais M, Kingston D, Giallo R, MacQueen GM, Giglia L, et al. Neonatal outcomes in women with untreated antenatal depression compared with women without depression: a systematic review and meta-analysis. JAMA Psychiatry. 2016;73(8):826-37.

19. Wouk K, Stuebe AM, Meltzer-Brody S. Post-delivery mental health and breastfeeding practices: an analysis using the 2010–2011 Pregnancy Risk Assessment Monitoring System. Matern Child Health J. 2017;21(3):636-47.

20. Howard LM, Kirkwood G, Latinovic R. Sudden infant death syndrome and maternal depression. J Clin Psychiatry. 2007;68(8):1279-83.

21. Sanderson CA, Cowden B, Hall DMB, Taylor EM, Carpenter RG, Cox JL. Is postnatal depression a risk factor for sudden infant death? Br J Gen Pract. 2002;52(481):636-40.

22. O’Donnell KJ, Glover V, Barker ED, O’Connor TG. The persisting effect of maternal mood in pregnancy on childhood psychopathology. Dev Psychopathol. 2014;26(2):393-403.

23. Dow-Fleisner S, Hawkins SS. Child physical well-being in the context of maternal depression. Soc Work Res. 2018 Jun;42(2):95-105.

24. Wojcicki JM, Holbrook K, Lustig RH, Epel E, Caughey AB, Muñoz RF, et al. Chronic maternal depression is associated with reduced weight gain in Latino infants from birth to 2 years of age. PLoS One. 2011;6(2):e16737.

25. Benton PM, Skouteris H, Hayden M. Does maternal psychopathology increase the risk of pre-schooler obesity? a systematic review. Appetite. 2015;87:259-82.

26. Cookson H, Granell R, Joinson C, Ben-Shlomo Y, Henderson AJ. Mothers’ anxiety during pregnancy is associated with asthma in their children. J Allergy Clin Immunol. 2009;123(4):847-53.e11.

27. Giallo R, Bahreinian S, Brown S, Cooklin A, Kingston D, Kozyrskyj A. Maternal depressive symptoms across early childhood and asthma in school children: findings from a longitudinal Australian population based study. PLoS One. 2015;10(3):e0121459.

28. Schwebel DC, Brezausek CM. Chronic maternal depression and children’s injury risk. J Pediatr Psychol. 2008;33(10):1108-16.

29. Yamaoka Y, Fujiwara T, Tamiya N. Association between maternal post-delivery depression and unintentional injury among 4-month-old infants in Japan. Matern Child Health J. 2016;20(2):326-36.

30. Flynn HA, Davis M, Marcus SM, Cunningham R, Blow FC. Rates of maternal depression in pediatric emergency department and relationship to child service utilization. Gen Hosp Psychiatry. 2004;26(4):316-22.

31. Sills MR, Shetterly S, Xu S, Magid D, Kempe A. Association between parental depression and children’s health care use. Pediatrics. 2007;119(4):e829-e836.

32. Minkovitz CS, Strobino D, Scharfstein D, Hou W, Miller T, Mistry KB, et al. Maternal depressive symptoms and children’s receipt of health care in the first 3 years of life. Pediatrics. 2005;115(2):306-14.

33. U.S. Bureau of Labor Statistics. Highlights of women's earnings in 2019. Washington (DC): U.S. Bureau of Labor Statistics; December 2020 [Cited 2021 Apr 15]. Available from: https://www.bls.gov/opub/reports/womens-earnings/2019/pdf/home.pdf

34. Shepard DS, Gurewich D, Lwin AK, Reed GA, Silverman MM. Suicide and suicidal attempts in the United States: costs and policy implications. Suicide Life Threat Behav. 2016;46:352-62.

35. Hao J, Hassen D, Hao Q, Graham J, Paglia MJ, Brown J, et al. Maternal and infant health care costs related to preeclampsia. Obstet Gynecol. 2019;134(6):1227-33.

36. Truven Health Analytics. The cost of having a baby in the United States, 2013. Greenwood Village (CO): Truven Health Analytics; 2013.

37. HCUP Fast Stats. Healthcare Cost and Utilization Project (HCUP). Rockville (MD): Agency for Healthcare Research and Quality; May 2020 [cited 2021 Apr 15].

38. Food and Nutrition Service. Supplemental Nutrition Assistance Program state activity report, FY 2016 [Internet]. Alexandria (VA): Food and Nutrition Service; 2017 [cited 2021 Apr 15]. Available from: https://fns-prod.azureedge.net/sites/default/files/snap/FY16-State-Activity-Report.pdf

39. Food and Nutrition Service. WIC program: total participation [Internet]. Alexandria (VA): Food and Nutrition Service; 2021 [cited 2021 Apr 15]. Available from: https://fns-prod.azureedge.net/sites/default/files/resource-files/26wifypart-1.pdf

40. Food and Nutrition Service. WIC program: food cost [Internet]. Alexandria (VA): Food and Nutrition Service; 2021 [cited 2021 Apr 15]. Available from: https://fns-prod.azureedge.net/sites/default/files/resource-files/24wicfood$-1.pdf

41. Food and Nutrition Service. WIC program: nutrition service and administrative costs [Internet]. Alexandria (VA): Food and Nutrition Service; 2021 [cited 2021 Apr 15]. Available from: https://fns-prod.azureedge.net/sites/default/files/resource-files/23WICAdm$-1.pdf

42. Centers for Medicare & Medicaid Services. Health expenditures by state of residence, 1991–2014. Baltimore (MD): Centers for Medicare & Medicaid Services; 2017 [cited 2021 Apr 15].

43. Kaiser Family Foundation. Medicaid spending per enrollee (full or partial Benefit), time-frame: FY 2014. Washington (DC): Kaiser Family Foundation; 2020 [cited 2021 Apr 15].

44. Office of Family Assistance. TANF financial data—FY 2016. Washington (DC): Office of Family Assistance; 2018 [cited 2021 Apr 15].

45. Office of Family Assistance. TANF caseload data 2016. Washington (DC): Office of Family Assistance; 2017 [cited 2021 Apr 15].

46. Waitzman NJ, Jalali A. Updating national preterm birth costs to 2016 with separate estimates for individual states [Internet]. Salt Lake City (UT): University of Utah; 2019 [cited 2021 Apr 15]. Available from: https://www.marchofdimes.org/peristats/documents/Cost_of_Prematurity_2019.pdf

47. Bartick M, Reinhold A. The burden of suboptimal breastfeeding in the united states: a pediatric cost analysis. Pediatrics. 2010;125(5):e1048-e1056.

48. Fox M, Cacciatore J, Lacasse JR. Child death in the United States: productivity and the economic impact of parental grief. Death Stud. 2014;38(6-10):597-602.

49. Beecham J. Annual research review: child and adolescent mental health interventions: a review of progress in economic studies across different disorders. J Child Psychol Psychiatry. 2014;55(6):714-32.

50. Finkelstein EA, Graham WCK, Malhotra R. Lifetime direct medical costs of childhood obesity. Pediatrics. 2014;133(5):854-62.

51. Sullivan PW, Ghushchyan V, Navaratnam P, Friedman HS, Kavati A, Ortiz B, et al. The national cost of asthma among school-aged children in the United States. Ann Allergy Asthma Immunol. 2017;119(3):246.e1-52.e1.

52. Centers for Disease Control and Prevention. Web-Based injury statistics query and reporting system of the National Center for Injury Prevention and Control [Internet]. Atlanta (GA): Centers for Disease Control and Prevention; 2019 [cited 2021 Apr 15]. Available from: https://webappa.cdc.gov/sasweb/ncipc/nfirates.html

53. Agency for Healthcare Research and Quality. Emergency room services—mean and median expenses per person with expense and distribution of expenses by source of payment: United States, 2014. Rockville (MD): Agency for Healthcare Research and Quality; 2019 [cited 2021 Apr 15].

54. Agency for Healthcare Research and Quality. MEPSnet household component for 2015 [Internet]. Rockville (MD): Agency for Healthcare Research and Quality; 2015 [cited 2021 Apr 15]. Available from: http://www.meps.ahrq.gov/mepsweb/data_stats/MEPSnetHC/saveselects_restore.action?ID=92e969c5-b5fe-4675-b6d6-9d75a940ca52&page=selrecord

55. Hamilton BE, Martin JA, Osterman MJK. Births: provisional data for 2019. Hyattsville (MD): National Center for Health Statistics; 2020 May [cited 2021 Apr 14]. Available from: https://www.cdc.gov/nchs/data/vsrr/vsrr-8-508.pdf

56. Curtin SC, Abma JC, Kost K. 2010 pregnancy rates among U.S. women [Internet]. Atlanta (GA): Centers for Disease Control and Prevention; 2015 [cited 2021 Apr 15]. Available from: https://www.cdc.gov/nchs/data/hestat/pregnancy/2010_pregnancy_rates.pdf

57. U.S. Census Bureau. Annual estimates of the resident population for selected age groups by sex for Texas: April 1, 2010 to July 1, 2019 [Internet]. Suitland (MD): U.S. Census Bureau; 2020 [cited 2021 Apr 15]. Available from: https://www.census.gov/data/tables/time-series/demo/popest/2010s-state-detail.html

58. Bauman BL, Ko JY, Cox S, D'Angelo DV, Warner L, Folger S, et al. Vital signs: post-delivery depressive symptoms and provider discussions about perinatal depression - United States, 2018. Morb Mortal Wkly Rep. 2020;69(19):575-81.

59. U.S. Bureau of Labor Statistics. Consumer price index for all urban consumers: medical care services in u.s. city average. [CUSR0000SAM2] [Internet]. St. Louis (MO): Federal Reserve Bank of St. Louis; 2021 [cited 2021 Feb 8]. Available from: https://fred.stlouisfed.org/series/CUSR0000SAM2

60. Sanders GD, Neumann PJ, Basu A, Brock DW, Feeny D, Krahn M, et al. Recommendations for conduct, methodological practices, and reporting of cost-effectiveness analyses: second panel on cost-effectiveness in health and medicine. JAMA. 2016;316(10):1093-103.

61. U.S. Bureau of Labor Statistics. Women in the labor force: a databook [Internet]. Washington (DC): U.S. Bureau of Labor Statistics; 2019 [cited 2021 Apr 15]. Available from: https://www.bls.gov/opub/reports/womens-databook/2019/pdf/home.pdf

62. Centers for Disease Control and Prevention. Underlying cause of death 1999–2018 on CDC WONDER Online Database [Internet]. Atlanta (GA): Centers for Disease Control and Prevention; 2020 [cited 2020 Dec 19]. Available from: http://wonder.cdc.gov/ucd-icd10.html

63. Fingar KR, Mabry-Hernandez I, Ngo-Metzger Q, Wolff T, Steiner CA, Elixhauser A. Delivery hospitalizations involving preeclampsia and eclampsia, 2005–2014 [Internet]. Rockville (MD): Agency for Healthcare Research and Quality; 2017 [cited 2021 Apr 15]. Available from: https://www.hcup-us.ahrq.gov/reports/statbriefs/sb222-Preeclampsia-Eclampsia-Delivery-Trends.pdf

64. Centers for Medicare and Medicaid Services. Fact sheet: Newborns' and Mothers' Health Protection Act of 1996 (NMHPA) [Internet]. Baltimore (MD): Centers for Medicare & Medicaid Services; n.d [2021 Feb 24]. Available from: https://www.cms.gov/CCIIO/Programs-and-Initiatives/Other-Insurance-Protections/nmhpa_factsheet

65. U.S. Census Bureau. Table S2201: Food stamps/Supplemental Nutrition Assistance Program (SNAP): 2019 ACS 1-year estimates subject tables [Internet]. Suitland (MD): U.S. Census Bureau; n.d. [cited 2021 Apr 15]. Available from: https://data.census.gov/cedsci/table?q=SNAP&t=-03%20-%20All%20available%20basic%20race%20combinations%3AFamilies%20and%20Household%20Characteristics%3AFamilies%20and%20Living%20Arrangements&g=0400000US48&tid=ACSST1Y2019.S2201&hidePreview=true

66. U.S. Department of Agriculture. National-level WIC coverage rates, by year and eligibility category, 2005–2017 [Internet]. Washington (DC): U.S. Department of Agriculture; 2018 [cited 2021 Apr 15]. Available from: https://www.fns.usda.gov/apps/WIC2019/data/national-wic-eligibility-and-participation-2005-2017.xlsx

67. Medicaid and CHIP Payment and Access Commission. Fact sheet: Medicaid's role in financing maternity care. Washington (DC): Medicaid and CHIP Payment and Access Commission; January 2020 [cited 2021 Apr 14]. Available from: https://www.macpac.gov/wp-content/uploads/2020/01/Medicaid%E2%80%99s-Role-in-Financing-Maternity-Care.pdf

68. Office of Family Assistance. Characteristics and financial circumstances of TANF recipients, fiscal year 2019 [Internet]. Washington (DC): Office of Family Assistance; 2020 [cited 2021 Apr 15]. Available from: https://www.acf.hhs.gov/ofa/data/characteristics-and-financial-circumstances-tanf-recipients-fiscal-year-2019

69. U.S. Census Bureau. American Community Survey, 2019 American Community Survey 1-year estimates, table S1702 [Internet]. Suitland (MD): U.S. Census Bureau; n.d [cited 2020 Dec 28]. Available from: https://data.census.gov/cedsci/table?q=families%20race%20&g=0400000US48&tid=ACSST1Y2019.S1702&hidePreview=false

70. Centers for Disease Control and Prevention. Breastfeeding report card: United States, 2020 [Internet]. Atlanta (GA): Centers for Disease Control and Prevention; 2020 [cited 2021 Apr 15]. Available from: https://www.cdc.gov/breastfeeding/data/reportcard.htm

71. National Center for Health Statistics. Linked birth / infant death records 2007–2018 [Internet]. Hyattsville (MD): National Center for Health Statistics; n.d. [cited 2020 Dec 15]. Available from: http://wonder.cdc.gov/lbd-current.html

72. Texas Medical Association. Mental health funding [Internet]. Austin (TX): Texas Medical Association; 2016 [cited 2021 Apr 15]. Available from: https://www.texmed.org/Template.aspx?id=19994

73. Robert Wood Johnson Foundation. Obesity rates among WIC participants ages 2–4 [Internet]. Princeton (NJ): Robert Wood Johnson Foundation; 2019 [cited 2021 Apr 15]. Available from: https://stateofchildhoodobesity.org/wic

74. Centers for Disease Control and Prevention. Table 4-1: current asthma prevalence percents by age, United States: National Health Interview Survey, 2018 [Internet]. Atlanta (GA): Centers for Disease Control and Prevention; 2019 [cited 2021 Apr 15]. Available from: https://www.cdc.gov/asthma/nhis/2018/table4-1.htm

75. Centers for Disease Control and Prevention. Nonfatal injury reports, 2000–2017 [Internet]. Atlanta (GA): Centers for Disease Control and Prevention; 2019 [cited 2021 Apr 15]. Available from: https://webappa.cdc.gov/sasweb/ncipc/nfirates.html

76. Rui P, Kang K. National Hospital Ambulatory Medical Care Survey: 2017 emergency department summary tables [Internet]. Hyattsville (MD): National Center for Health Statistics; 2017 [cited 2021 Apr 15]. Available from: https://www.cdc.gov/nchs/data/nhamcs/web_tables/2017_ed_web_tables-508.pdf

77. Wolf ER, Hochheimer CJ, Sabo RT, DeVoe J, Wasserman R, Geissal E, et al. Gaps in well-child care attendance among primary care clinics serving low-income families. Pediatrics. 2018;142(5):e20174019.

78. Texas Health and Human Services Commission. 2019 healthy Texas mothers & babies data book [Internet]. Austin (TX): Texas Health and Human Services Commission; 2019 [cited 2021 Apr 15]. Available from: https://www.dshs.state.tx.us/healthytexasbabies/Documents/HTMB-Data-Book-2019-20200206.pdf

79. U.S. Census Bureau. Annual state resident population estimates for 5 race groups (5 race alone or in combination groups) by age, sex, and Hispanic origin: April 1, 2010 to July 1, 2019. (SC-EST2019-ALLDATA5) [Internet]. Suitland (MD): U.S. Census Bureau; 2020 [cited 2021 Feb 24]. Available from: https://www2.census.gov/programs-surveys/popest/tables/2010-2019/state/asrh/sc-est2019-alldata5.csv

80. Martin JA, Hamilton BE, Osterman MJK. Births in the United States, 2019 [Internet]. Hyattsville (MD): National Center for Health Statistics; 2020 [cited 2021 Apr 15]. Available from: https://www.cdc.gov/nchs/data/databriefs/db387-H.pdf

81. Anstey EH, Chen J, Elam-Evans LD, Perrine CG. Racial and geographic differences in breastfeeding—United States, 2011–2015. Morb Mortal Wkly Rep. 2017;66(27).

82. Foster BA, Maness TM, Aquino CA. Trends and disparities in the prevalence of childhood obesity in South Texas between 2009 and 2015. J Obes. 2017;2017.
